# Airway macrophage-intrinsic TGF-β1 regulates pulmonary immunity during early-life allergen exposure

**DOI:** 10.1016/j.jaci.2021.01.026

**Published:** 2021-05

**Authors:** William J. Branchett, James Cook, Robert A. Oliver, Nicoletta Bruno, Simone A. Walker, Helen Stölting, Matthias Mack, Anne O’Garra, Sejal Saglani, Clare M. Lloyd

**Affiliations:** a National Heart and Lung Institute, Faculty of Medicine, Imperial College London, London, United Kingdom; b Department of Paediatric Respiratory Medicine, Royal Brompton Hospital, London, United Kingdom; c Department of Internal Medicine II- Nephrology, University Hospital Regensburg, Regensburg, Germany; d Laboratory of Immunoregulation and Infection, The Francis Crick Institute, London, United Kingdom

**Keywords:** Asthma, type 2 immunity, neonate, TGF-β, lung immunity, macrophage, monocyte, chemokine, AAD, Allergic airway disease, αCCR2, Anti-CCR2, αCCR8, Anti-CCR8, AM, Airway macrophage, BAL, Bronchoalveolar lavage, cDC, Conventional dendritic cell, HDM, House dust mite, ILC2, Group 2 innate lymphoid cell, IM, Interstitial macrophage, mLN, Mediastinal lymph node, MP, Mononuclear phagocyte, P, Postnatal day, T2, Type 2

## Abstract

**Background:**

Early life represents a major risk window for asthma development. However, the mechanisms controlling the threshold for establishment of allergic airway inflammation in early life are incompletely understood. Airway macrophages (AMs) regulate pulmonary allergic responses and undergo TGF-β–dependent postnatal development, but the role of AM maturation factors such as TGF-β in controlling the threshold for pathogenic immune responses to inhaled allergens remains unclear.

**Objective:**

Our aim was to test the hypothesis that AM-derived TGF-β1 regulates pathogenic immunity to inhaled allergen in early life.

**Methods:**

Conditional knockout (*Tgfb1*^ΔCD11c^) mice, with TGF-β1 deficiency in AMs and other CD11c^+^ cells, were analyzed throughout early life and following neonatal house dust mite (HDM) inhalation. The roles of specific chemokine receptors were determined by using *in vivo* blocking antibodies.

**Results:**

AM-intrinsic TGF-β1 was redundant for initial population of the neonatal lung with AMs, but AMs from *Tgfb1*^ΔCD11c^ mice failed to adopt a mature homeostatic AM phenotype in the first weeks of life. Evidence of constitutive TGF-β1 signaling was also observed in pediatric human AMs. TGF-β1–deficient AMs expressed enhanced levels of monocyte-attractant chemokines, and accordingly, *Tgfb1*^ΔCD11c^ mice exposed to HDM throughout early life accumulated CCR2-dependent inflammatory CD11c^+^ mononuclear phagocytes into the airway niche that expressed the proallergic chemokine CCL8. *Tgfb1*^ΔCD11c^ mice displayed augmented T_H_2, group 2 innate lymphoid cell, and airway remodeling responses to HDM, which were ameliorated by blockade of the CCL8 receptor CCR8.

**Conclusion:**

Our results highlight a causal relationship between AM maturity, chemokines, and pathogenic immunity to environmental stimuli in early life and identify TGF-β1 as a key regulator of this.

## Graphical abstract

**Figure:**
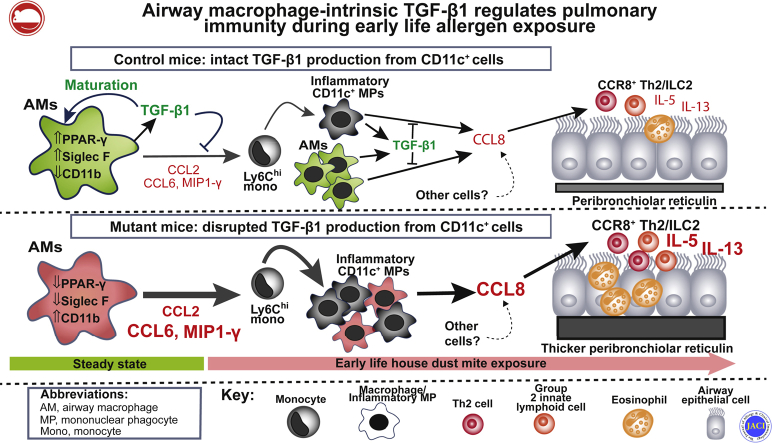

The immune system is highly dynamic in early life^1^^,^^2^ and distinct from that in adults. Exposure to airborne pathogens and aeroallergens typically occurs rapidly after birth, yet neonatal immunity is characterized by relatively weak innate responses to infection^3^ and a type 2 (T2) immune bias.^4^ This neonatal period therefore represents a major risk window for allergic sensitization and development of asthma, a disease that is very common in childhood.^4^^,^^5^ The distinct immunologic landscape in early life necessitates greater understanding of how specific features of the developing neonatal lung influence allergic immunity and development of asthma.^5^

The neonatal period is crucial for the seeding and maturation of resident airway macrophages (AMs). AMs are phenotypically distinct lung-resident macrophages that differentiate from their fetal precursors in the first week of life. Once matured, AMs are marked by high expression of CD11c and, in mice, by high expression of Siglec F, along with pan-macrophage markers such as F4/80 and CD64, but little to no steady-state CD11b expression.^6^ This profile distinguishes AMs from lung interstitial macrophages (IMs), which express the macrophage markers CD11b^+^, F4/80^+^, and CD64^+^ but lack Siglec F and express little to no CD11c.^7^

AMs are important sentinels of pulmonary homeostasis,^8^ and AM depletion in adult mice enhances experimental allergic airway disease (AAD) severity, which is consistent with an intrinsic capacity of AMs to dampen allergic responses.^9^^,^^10^ However, AMs also produce mediators associated with asthma pathogenesis in patients and experimental AAD,^11^, ^12^, ^13^, ^14^, ^15^ whereas dysfunctional AMs can drive T2 immunity and pathogenic tissue repair responses to house dust mite (HDM) allergen.^12^ Although prenatally derived AMs self-renew in adulthood,^16^ inflammatory monocytes can differentiate into AMs during allergic inflammation, influenza infection, and bleomycin lung injury in adult mice^17^, ^18^, ^19^ and gradually replace prenatally derived AMs in the steady state with advancing age.^20^^,^^21^ Recruited monocytes can also differentiate into non-AM inflammatory mononuclear phagocyte (MP) populations that are thought to act locally to promote allergic inflammation in the lung.^10^^,^^18^^,^^22^ Recruited MPs, along with resident AMs, are therefore potentially pivotal cells during AAD, but the factors governing the balance between resident and recruited MPs and their phenotypes in disease are incompletely understood.

TGF-β1 is a pleiotropic regulator of immune responses alongside other diverse cellular functions.^23^ AMs are a major source of pulmonary TGF-β1, and TGF-β signaling to AMs is essential throughout their postnatal development.^24^ However, the effects of AM-intrinsic TGF-β1 signaling on AM development throughout early life and its implications for immune regulation are not yet clear.

Herein, we have examined the role of AM-derived TGF-β1 throughout AM development and in the context of neonatal exposure to aeroallergens. AM-intrinsic TGF-β1 expression was required for maintenance and maturation of murine AMs over the first 4 weeks of but not for their initial population of the lung, which is in contrast to the findings of previous reports of mice lacking all TGF-β signaling to AMs.^24^ Evidence of constitutive TGF-β1 imprinting of identity was also observed in AMs from children. Mice with TGF-β1 deficiency in CD11c^+^ cells displayed dysregulated chemokine production and enhanced inflammatory MP responses to neonatal allergen exposure, along with more severe AAD. This work highlights the importance of tissue- and age-specific factors in imprinting resident macrophage phenotypes to facilitate immune homeostasis during the critical early-life window of immune maturation and initial environmental exposures.

## Methods

Additional experimental procedures are described in the Methods section of the Online Repository (available at www.jacionline.org).

### Mice

All animal work was performed in accordance with the Imperial College guidelines for the use of laboratory animals and the UK Animals (Scientific Procedures) Act 1986, under project license P996A24E1. *Tgfb1*^ΔCD11c^ conditional knockout mice were bred by mating *Itgax*-Cre^25^ and *Tgfb1*^fl/fl^^26^ mice and maintained as Cre hemizygous knockouts and Cre-negative *Tgfb1*^fl/fl^ littermate controls. C57BL/6J mice for bone marrow–derived macrophage generation were purchased from Charles River Laboratories.

### Early-life AAD model and chemokine receptor blockade

The mice received 3 intranasal installations of 20 μg (by total protein) of HDM extract (Greer Laboratories, Lenoir, NC) in 10 μL of sterile PBS (Gibco, Thermo Fisher, Waltham, Mass), without anaesthesia, from days 7 to 12 of life. A further 6 intranasal challenges of 25 μg of HDM extract in 15 μL of PBS were administered from days 14 to 27 of life under light isoflurane anaesthesia. HDM challenges were spaced 1 to 2 days apart 3 times per week, and equivalent volumes of PBS were used as controls where applicable. The mice were weaned at 20 to 23 days of age. In some experiments, rat anti-mouse blocking antibodies to CCR2 (clone MC-21,^27^ Matthias Mack Laboratory; 10 μg/dose), CCR8 (clone SA214G2, BioLegend, San Diego, Calif; 3 μg/dose), or equivalent doses of appropriate IgG2bκ isotype control antibodies (clone MC-67, Matthias Mack Laboratory and clone RTK4530, BioLegend for CCR2 and CCR8, respectively) were administered by intraperitoneal injection in a volume of 100 μL of PBS 24 hours before the seventh HDM challenge and then immediately before the seventh, eighth, and ninth HDM challenges. All analysis was performed 24 hours after the final HDM exposure.

### AM isolation from pediatric human subjects

The children were recruited prospectively between September 2015 and August 2018 at the Royal Brompton Hospital, London, United Kingdom, with informed consent from their parents. All work with human subjects was approved by the Hampstead Research Ethics Committee (reference no., 15/LO/1885; project title, Patterns of Airway Infection and Inflammation in Children with Respiratory Symptoms), permitting use of surplus lower airway samples from clinical investigations for research purposes. Children undergoing clinically indicated flexible bronchoscopy as part of investigation for either severe preschool wheeze or chronic recurrent wet cough were included in the present study. Preschool wheeze was defined as 3 or more episodes of wheeze, including 2 or more in the previous 6 months. Subjects were excluded from the study if diagnosed with cystic fibrosis, primary ciliary dyskinesia, congenital heart disease, or structural lung abnormalities, and all of them had been free of symptomatic respiratory infection for at least 2 weeks before bronchoscopy. Further patient details are presented in Table E1 (in this article's Online Repository at www.jacionline.org).

Bronchoalveolar lavage (BAL) within the right middle lung lobe was performed while the children were under general anaesthesia; it was performed by using 3 volumes of 0.9% saline (each at a rate of 1 mL/kg of body weight). BAL fluid differential cell counts, bacterial culture, and viral PCR (using a multiplex respiratory pathogens kit from Fast Track Diagnostics) were performed according to routine diagnostic practice at the Royal Brompton Hospital Pathology Department. BAL fluid was filtered through 70-μm cell strainers. The cells were pelleted before brief incubation in ammonium chloride erythrocyte lysis buffer and suspension in complete RPMI medium. Up to 10^7^ cells were incubated in magnetic activated cell sorting buffer (0.5 % FBS and 2 mM EDTA in PBS) containing allophycocyanin/cyanine 7–conjugated anti-human CD206 antibody (BioLegend, clone 15-2) in the presence of Human TruStain FcX Fc-receptor–blocking reagent (BioLegend), for 10 minutes at 4^o^C, before binding of anti–cyanine 7 microbeads (Miltenyi Biotec, Bergisch Gladbach, Germany) at 4^o^C for 15 minutes and magnetic positive selection of labeled cells using LS separator columns, a midiMACS magnetic separator, and MACS Multi-Stand (all obtained from Miltenyi Biotec), as per the manufacturer’s instructions. The purity of the positive AM fractions was confirmed to be higher than 90% by flow cytometry in all samples.

### Upstream regulator analysis of RNA-Seq data

Upstream regulator prediction was performed by using Ingenuity Pathway Analysis (IPA, QIAGEN, Hilden, Germany) on the 200 genes with the highest median expression by RNA sequencing (RNA-Seq) in AMs from all study subjects. Both direct and indirect relationships were considered, and the results were limited to cytokines and growth factors. Genes identified as TGF-β1–regulated were overlaid with TGF-β1 by using the IPA Path Designer tool, and reported “expression” and “transcription” connections between genes were visualized. The literature reporting these connections was then manually inspected and gene symbols were color-coded based on positive, negative, or both positive and negative regulation by TGF-β1.

### Statistical analysis

Statistical testing and graphical representation of nontranscriptomic data were performed by using Prism, version 8, software (GraphPad Software, San Diego, Calif). Normally and nonnormally distributed data were represented by means and medians, respectively. Normally distributed data were analyzed by using Student *t* tests and ANOVA with Sidak *post hoc* tests for individual and multiple comparisons, respectively. Data that were not normally distributed were analyzed by using Mann-Whitney *U* tests and Kruskal-Wallis with Dunn *post hoc* tests for individual and multiple comparisons, respectively. Two-tailed tests were used. The figure legends describe the specific tests and results for each experiment. Statistical significance was determined as a *P* value less than .05.

### Data availability statement

The RNA sequencing data supporting this study have been deposited in the Gene Expression Omnibus and are available at series GSE144033. The other data sets supporting this study are available from the corresponding author on request.

## Results

### TGF-β1 from CD11c^+^ cells is required for maturation and maintenance of AMs in early life but not for their initial population of the lung

**Fig 1:**
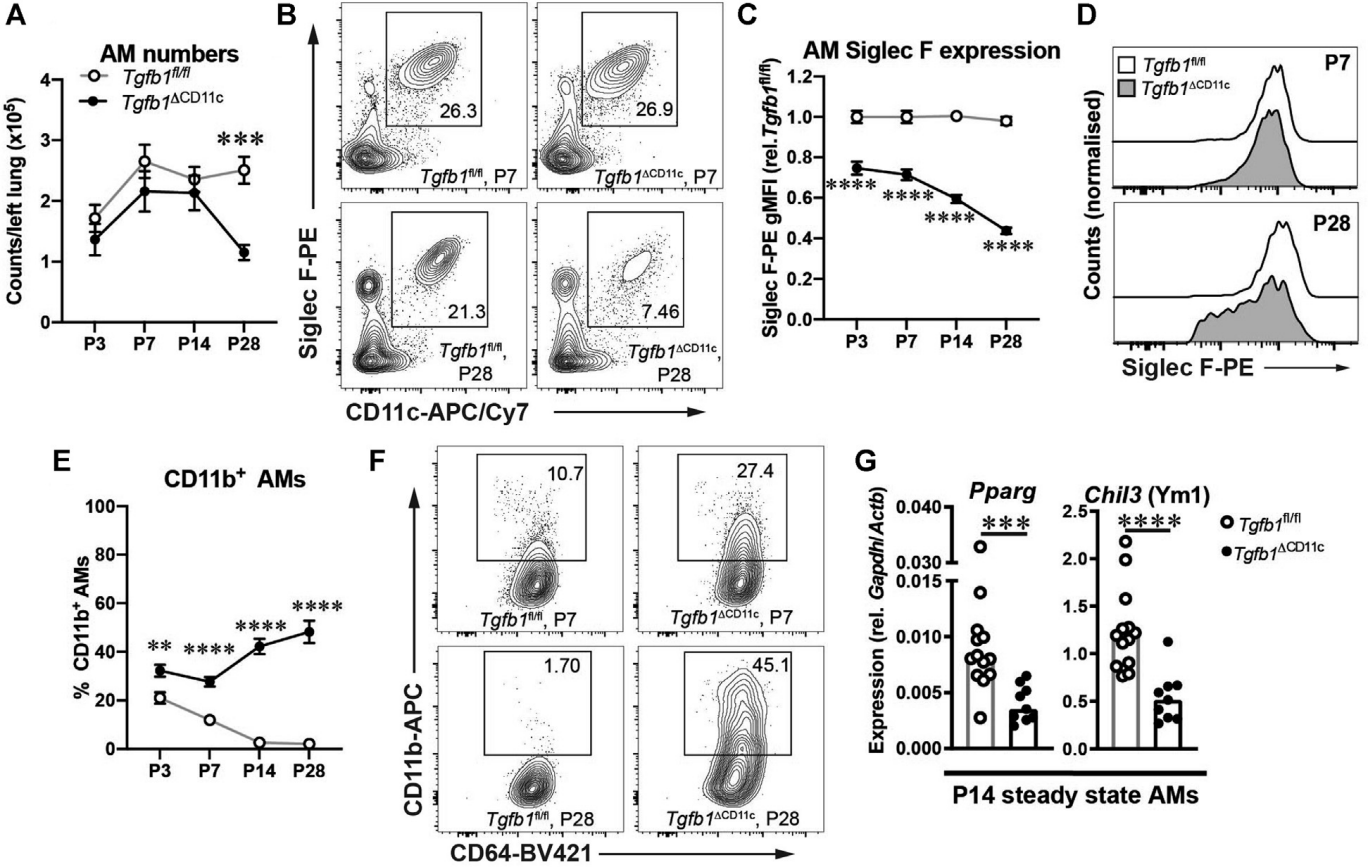
CD11c^+^ cell–derived TGF-β1 is required for AM maturation and maintenance in early life, but not for initial appearance of AMs in the lung. **A-F,** Lung cells from *Tgfb1*^ΔCD11c^ and *Tgfb1*^fl/fl^ mice were analyzed by flow cytometry at the indicated ages. **A**, AM numbers. **B**, Representative plots showing CD11c^+^Siglec F^+^ AMs gated from CD45^+^Lin^–^ (non-B/T/innate lymphoid cell/natural killer) cells. **C**, Geometric mean fluorescence intensity of Siglec F staining on AMs shown relative to that in the *Tgfb1*^fl/fl^ controls at each time point. **D**, Representative histograms showing Siglec F staining on AMs. **E**, Percentages of CD11b^+^ AMs. **F**, Representative plots showing CD11b expression on AMs. **G,** Expression of the indicated genes by quantitative PCR in AMs isolated from P14 *Tgfb1*^ΔCD11c^ and *Tgfb1*^fl/fl^ mice. Data are pooled from analysis of a minimum of 2 litters and are presented as means with either SEs or all individual replicates. In (**A**), (**C**), and (**E**), *Tgfb1*^fl/fl^ points show data from 8 (P3), 16 (P7), 18 (P14), and 10 (P28) mice, and *Tgfb1*^ΔCD11c^ points show data from 8 (P3), 11 (P7), 7 (P14), and 7 (P28) mice. Statistical results shown are from unpaired Student *t* tests: ∗∗*P* < .01; ∗∗∗*P* < .001; ∗∗∗∗*P* < .0001. *APC*, Allophycocyanin; *Cy7*, cyanine 7; *PE*, phycoerythrin; *rel.*, relative to.

**Fig E1:**
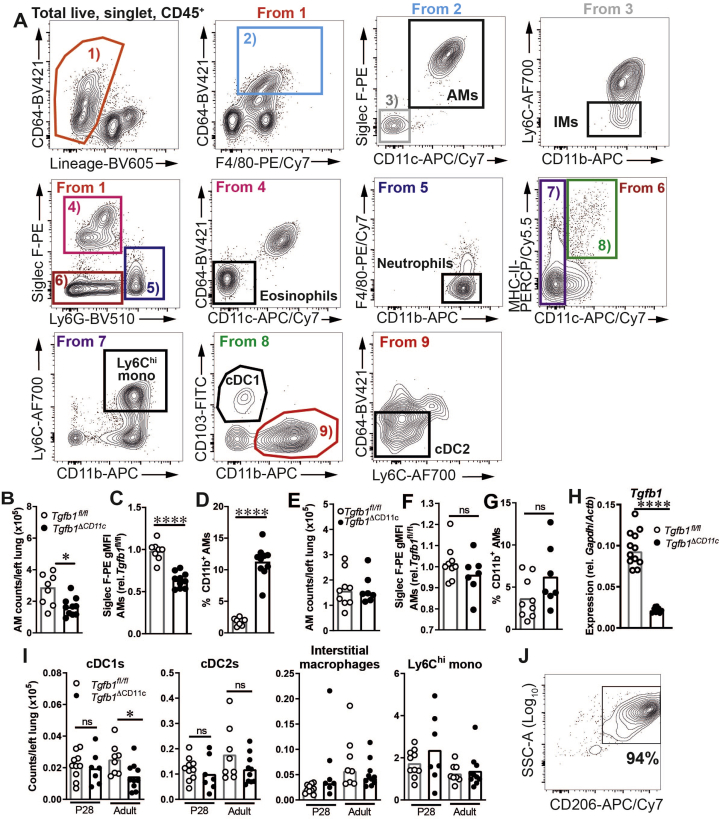


To examine the effects of deficiency in AM-intrinsic TGF-β1 production on pulmonary MP populations throughout the crucial first weeks of life, lung cells from CD11c-conditional TGF-β1 knockout (*Tgfb1*^fl/fl^ × CD11c-Cre, hereafter *Tgfb1*^ΔCD11c^) mice were analyzed by flow cytometry (see Fig E1, *A* in the Online Repository at www.jacionline.org). CD11c^+^Siglec F^+^ AMs were present at postnatal day 3 (P3) in *Tgfb1*^fl/fl^ littermate control mice, and their numbers remained similar from P7 to adulthood (8-10 weeks [Fig 1, *A* and see Fig E1, *B*]). Consistent with previous findings,^24^ AM numbers were decreased by more than 50 % in *Tgfb1*^ΔCD11c^ mice at P28 (Fig 1, *A*). However, our longitudinal analysis revealed that AM numbers were comparable between the *Tgfb1*^ΔCD11c^ and control mice over the first crucial postnatal weeks from P3 to P14 (Fig 1, *A*), which is suggestive of an age-dependent effect of intrinsic TGF-β1 deficiency on AM numbers that was not previously reported.

Despite their equivalent numbers at P3 to P14, *Tgfb1*^ΔCD11c^ mouse AMs displayed an altered phenotype even at P3. Although control AMs were uniformly Siglec F^hi^ and lost CD11b expression by P14, the *Tgfb1*^ΔCD11c^ mouse AMs remained Siglec F^+^ but had a consistently lower Siglec F staining intensity than that of their age-matched littermate controls (Fig 1, *C* and *D*), and a portion of them retained expression of CD11b, which increased further from P14 to P28 (Fig 1, *E* and *F*). Gene expression analysis at P14 confirmed the substantial reduction in *Tgfb1* gene expression in bulk *Tgfb1*^ΔCD11c^ mouse AMs, accompanied by reduced expression of the key AM transcription factor *Pparg*/PPAR-γ,^28^ which has previously been shown to require TGF-β1 signaling for maximal expression (Yu et al^24^), as well as the hallmark AM gene *Chil3*/Ym1^17^ (Fig 1, *G* and see Fig E1, *H*). These data were consistent with TGF-β1–deficient AMs failing to fully adopt the homeostatic AM phenotype during this early-life window.

AM numbers remained reduced in young adult *Tgfb1*^ΔCD11c^ mice, as did decreased Siglec F expression intensity and an increased proportion of CD11b^+^ AMs. However, these changes were less marked than at P28 (see Fig E1, *B*-*D*), suggesting that the AM deficiency observed in juvenile *Tgfb1*^ΔCD11c^ mice is not stable throughout life. Accordingly, no significant differences in AM number or Siglec F and CD11b expression were observed in 8- to 10-month-old *Tgfb1*^ΔCD11c^ mice (see Fig E1, *E-G*), and no illness was observed throughout the lives of these mice, with lung function and histology comparable to those in the controls when assessed in the mice at 7 months of age (data not shown). Importantly, early-life defects in the steady-state pulmonary MP compartment of *Tgfb1*^ΔCD11c^ mice were restricted to AMs, with comparable numbers of conventional dendritic cells (cDCs), Siglec F^–^CD11c^–^CD11b^+^Ly6C^–^ IMs and Ly6C^hi^ monocytes to *Tgfb1*^fl/fl^ controls at P28, although a small decrease in CD103^+^ type 1 cDC counts was apparent in young adult knockouts (see Fig E1, *I*).

Thus, although redundant for initial generation of CD11c^+^Siglec F^+^ AMs in the neonatal lung, TGF-β1 expression in CD11c^+^ cells was required for AM maintenance into late neonatal development and adoption of the classical airway-resident profile, revealing dynamic effects of TGF-β1 during this critical developmental window.

### Evidence of constitutive regulation of pediatric human AM identity by TGF-β1

**Fig 2:**
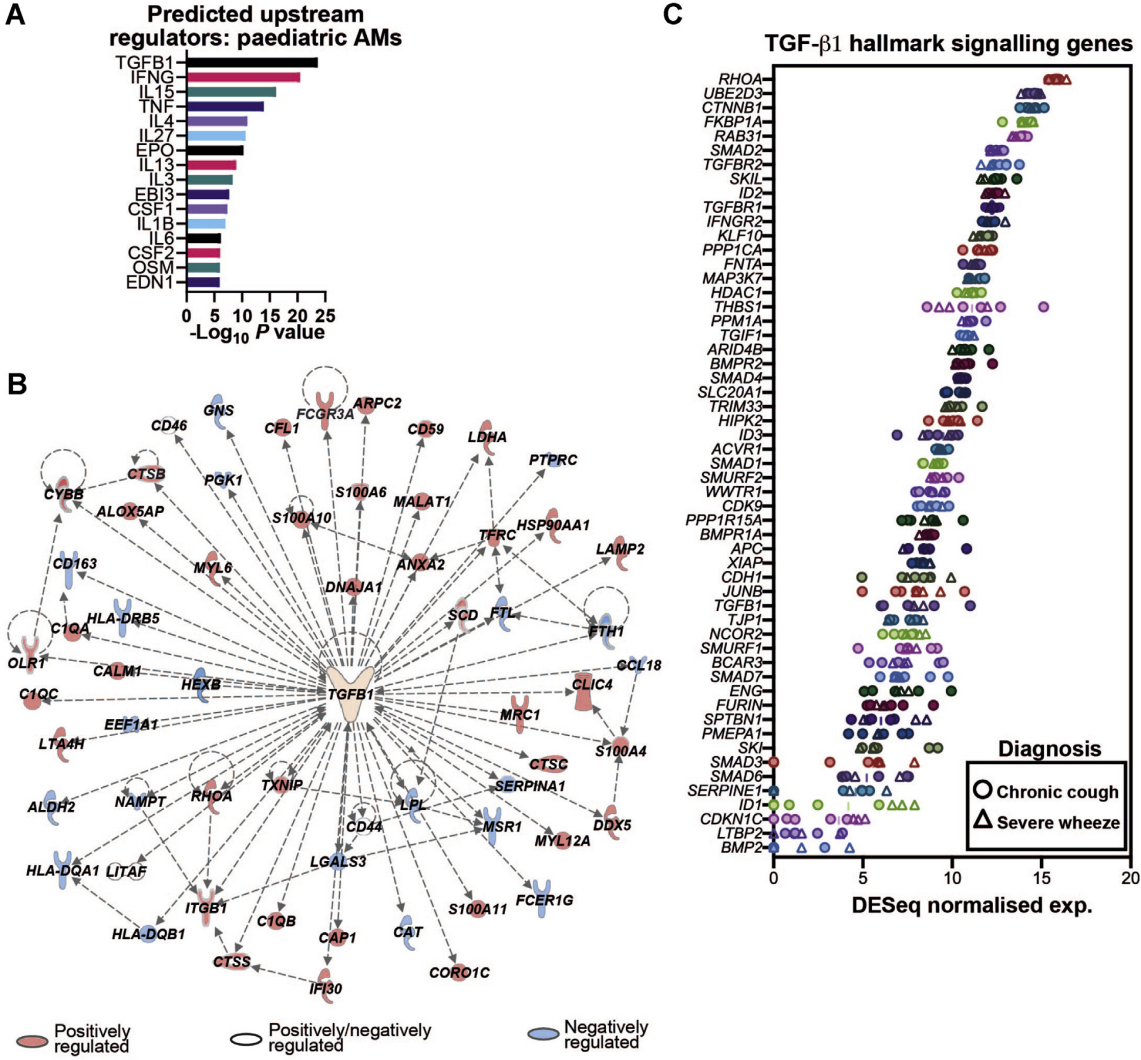
TGF-β1–induced genes are highly expressed in human pediatric AMs. RNA-Seq analysis of CD206^+^ AMs from BAL fluid of 8 pediatric subjects under investigation for either severe preschool wheeze (n = 3) or chronic cough (n = 5). **A,** Cytokine and growth factor regulators upstream of the 200 most highly expressed genes in all subjects predicted by using Ingenuity Pathway Analysis. Fisher exact *P* values are shown. **B,** Gene interaction network showing TGF-β1–regulated genes from (**A**) that have been color-coded according to reported positive and/or negative regulation by TGF-β1 in the Ingenuity Knowledge Base. Arrows denote direction of effects on expression. **C,** Normalized RNA-Seq expression values of the Broad Institute MSigDB HALLMARK_TGF_BETA_SIGNALING gene set in pediatric AM samples. Symbol shapes indicate patient diagnosis. Means and individual replicate values are shown. *exp*, Expression.

To determine whether TGF-β1 is also likely to imprint human AM identity in early life, we performed RNA-Seq on AMs sorted from BAL fluid of children under investigation for either chronic recurrent cough or severe preschool wheeze (see Fig E1, *J* and Table E1). The subjects had no symptomatic respiratory tract infection at the time of sampling, although some had detectable bacteria and/or viruses in their BAL fluid (see Table E1). Ingenuity's Upstream Regulator Analysis was performed on the 200 most highly expressed genes across all patients (irrespective of diagnosis), identifying TGF-β1 as the most enriched upstream cytokine or growth factor of highly expressed genes in pediatric AMs, with the majority (40 of 60) of the TGF-β1–regulated genes in this set reported as positively TGF-β1–regulated (Fig 2, *A* and *B*). Accordingly, pediatric AMs also expressed high levels of *TGFB1* and TGF-β canonical signaling pathway genes, with *RHOA* showing the highest expression among the canonical TGF-β–induced genes in pediatric AMs (Fig 2, *C*). These data suggest that TGF-β1 constitutively regulates human, as well as murine, AM gene expression in early life.

### *Tgfb1*^ΔCD11c^ mice accumulate inflammatory MPs in the lungs following neonatal allergen exposure

**Fig 3:**
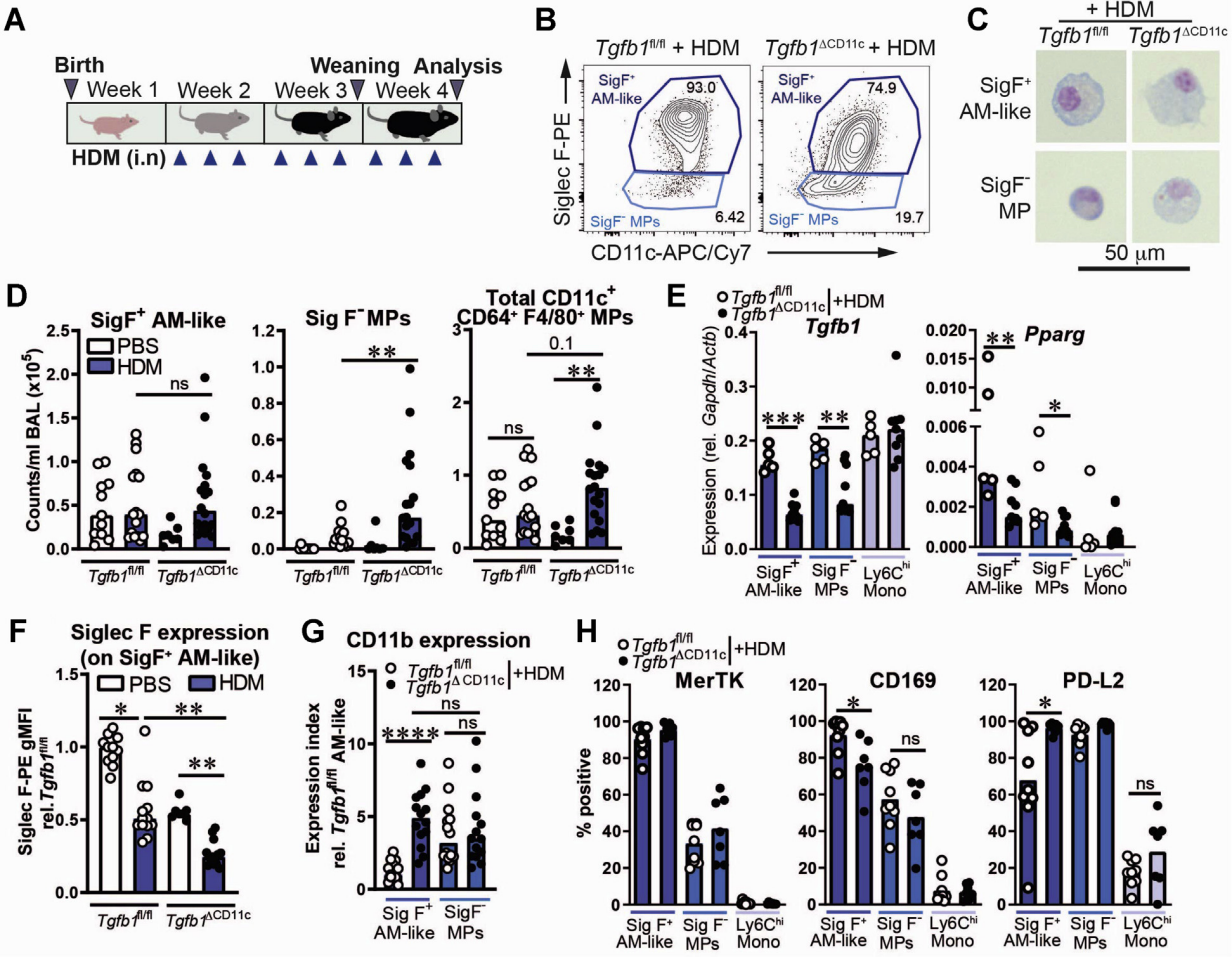
Inflammatory CD11c^+^ MPs accumulate following neonatal HDM exposure in *Tgfb1*^ΔCD11c^ mice. **A,** Neonatal AAD model schematic. **B-H,** Flow cytometry analysis of pulmonary MP populations. **B,** Representative plots showing subpopulations of CD64^+^ F4/80^+^ MPs in BAL fluid after HDM treatment, as distinguished on the basis of Siglec F expression (SigF^+^ or SigF^-^ when referring to these subpopulations). **C,** Representative images of cells from populations in (**B**). **D,** Numbers of SigF^+^ AM-like cells, SigF^–^ MPs, and combined populations in BAL fluid. **E,** Relative quantitative PCR gene expression in sorted MP populations in BAL fluid. **F,** Geometric mean fluorescence intensity of Siglec F staining on AMs/SigF^+^ AM-like cells relative to the *Tgfb1*^fl/fl^/PBS group. **G,** CD11b staining on BAL fluid CD11c^+^ MP populations. **H,** Percentages of BAL fluid MP populations with positive staining for the indicated surface markers compared with lung Ly6C^hi^ monocytes (Mono). Scatter plots and bar plots show means or medians of data pooled from 2 to 4 individual experiments, with all individual replicates. Statistical results shown are from 1-way ANOVA with the Sidak *post hoc* test for multiple comparisons and either Student unpaired *t* tests or Mann-Whitney *U* tests for single comparisons. ∗*P* < .05; ∗∗*P* < .01; ∗∗∗*P* < .001; ∗∗∗∗*P* < .0001. *APC*, Allophycocyanin; *Cy7*, cyanine 7; *i.n*, intranasal; *ns*, nonsignificant; *PE*, phycoerythrin.

Because AMs are key immune-regulatory cells and *Tgfb1*^ΔCD11c^ mice showed markedly impaired AM maturation between 1 to 4 weeks of life, we hypothesized that this perturbed lung MP state would result in a dysregulated pulmonary MP response to neonatal allergen exposure. We therefore repeatedly administered intranasal HDM to *Tgfb1*^ΔCD11c^ mice and littermate *Tgfb1*^fl/fl^ control mice throughout this early-life window from P7 to P28 (Fig 3, *A*) and analyzed MP populations by flow cytometry (Fig 3, *B* and see Fig E1, *A*). In contrast to allergen-naive mice, in which all airway CD11c^+^F4/80^+^ cells are Siglec F^+^ AMs,^6^ 2 major populations of CD11c^+^CD64^+^F4/80^+^ macrophage-like MPs could be distinguished in the BAL fluid of P28 mice after 3 weeks of HDM exposure on the basis of Siglec F expression. One population expressed Siglec F at variable levels and fell into the AM gate defined at steady state, whereas the other, which was not apparent at steady state, lacked detectable Siglec F expression by flow cytometry (Fig 3, *B*). The large size and AM-like morphology of the Siglec F^+^ population (Fig 3, *C*) suggested that this population comprised *bona fide* AMs and/or newly recruited cells adopting an AM-like phenotype; as a result, they were termed *SigF*^*+*^ *AM-like cells*. In contrast, the Siglec F^–^CD11c^+^ population (henceforth referred to as *SigF*^*–*^ *MPs*) displayed a macrophage-like morphology but were comparatively smaller than AMs (Fig 3, *C*), which was suggestive of non-AM–recruited MPs.

**Fig E2:**
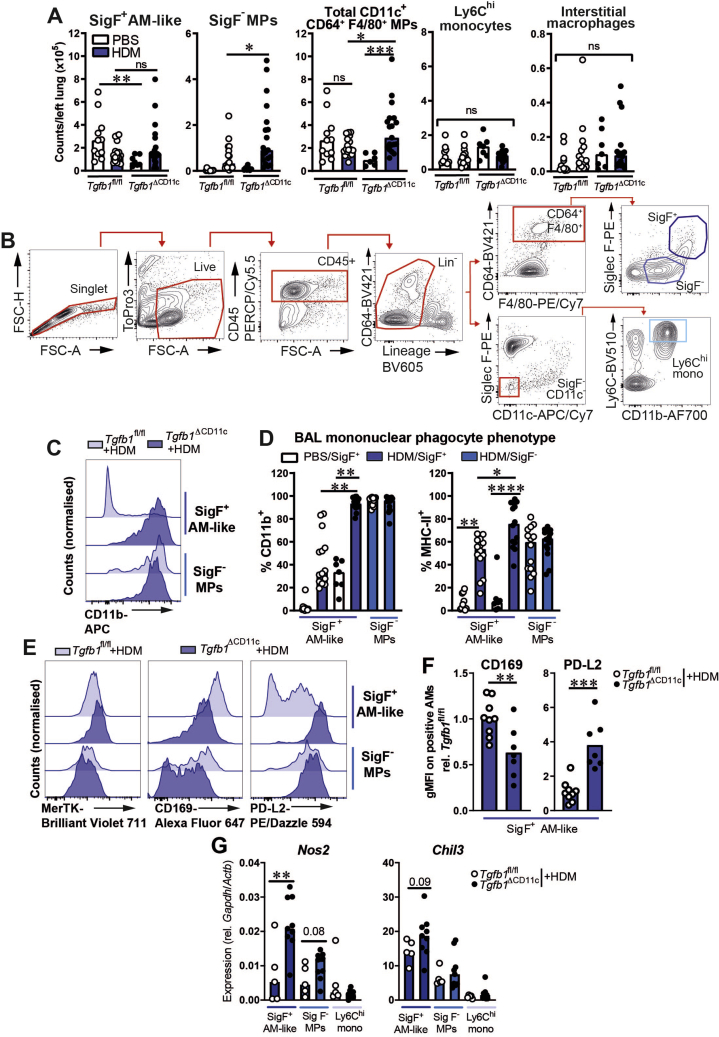


As in the lungs of naive P28 mice (Fig 1, *A*), AM numbers were reduced in the BAL fluid and lung tissue of P28 *Tgfb1*^ΔCD11c^ mice after 3 weeks of PBS mock treatment (Fig 3, *D* and see Fig E2, *A* in the Online Repository at www.jacionline.org). However, HDM-treated *Tgfb1*^ΔCD11c^ mice displayed numbers of SigF^+^ AM-like cells comparable to the numbers of those in their *Tgfb1*^fl/fl^ littermates (Fig 3, *D* and see Fig E2, *A*), suggesting that HDM exposure drove either persistence of TGF-β1–deficient resident AMs or recruitment of replacement AM-like cells to the lung. Furthermore, compared with the *Tgfb1*^fl/fl^ controls, the *Tgfb1*^ΔCD11c^ mice displayed enhanced accumulation of SigF^–^ MPs in the BAL fluid and lungs, resulting in an increase in the number of total pulmonary CD11c^+^CD64^+^F4/80^+^ MPs in *Tgfb1*^ΔCD11c^ mice but not in their littermate controls, following repeated HDM exposure (Fig 3, *D* and see Fig E2, *A*). Sorted SigF^+^ AM-like cells and SigF^–^ MPs (see Fig E2, *B*) had diminished *Tgfb1* mRNA expression, which is consistent with Cre-mediated *Tgfb1* deletion being maintained within these CD11c^+^ populations during allergic inflammation, concomitant with decreased *Pparg* expression (Fig 3, *E*). In contrast to the numbers of CD11c^+^ MPs, no changes were observed in the numbers of Ly6C^hi^ monocytes or IMs (see Fig E2, *A*).

Despite their equivalent numbers in HDM-treated *Tgfb1*^fl/fl^ and *Tgfb1*^ΔCD11c^ mice, the SigF^+^ AM-like cells from *Tgfb1*^ΔCD11c^ mice displayed a distinct, more proinflammatory, phenotype. Although the SigF^+^ AM-like cells from HDM-treated mice of both genotypes had a reduced Siglec F staining intensity as compared with that of the steady-state AMs from their respective PBS-treated controls, SigF^+^ AM-like cells from HDM-treated *Tgfb1*^ΔCD11c^ mice displayed the lowest expression of all the groups (Fig 3, *B* and *F*). CD11b, a marker of activation of mature AMs,^29^ was also more highly expressed on SigF^+^ AM-like cells from HDM-treated *Tgfb1*^ΔCD11c^ mice than on SigF^+^ AM-like cells from the *Tgfb1*^fl/fl^ mice, with nearly 100% expressing CD11b in the *Tgfb1*^ΔCD11c^ mice (Fig 3, *G* and see Fig E2, *C* and *D*). In contrast, SigF^–^ MPs were almost all CD11b^+^, with comparable expression between *Tgfb1*^ΔCD11c^ and *Tgfb1*^fl/fl^ mice (Fig 3, *G* and see Fig E2, *C* and *D*). Similarly, a greater proportion of SigF^+^ AM-like cells were MHC class II^+^ in *Tgfb1*^ΔCD11c^ than *Tgfb1*^fl/fl^ mice, whereas MHC class II was expressed by 40% to 80% of SigF^–^ MPs in both groups (see Fig E2, *D*). Consistent with their definition as AM-like, nearly all SigF^+^ AM-like cells expressed the pan-macrophage marker MerTK, which was absent from Ly6C^hi^ monocytes (Fig 3, *H* and see Fig 2, *E*). However, only a subset of SigF^–^ MPs were MerTK^+^ (Fig 3, *H* and see Fig E2, *E*), which was suggestive of a heterogeneous inflammatory population that includes a large proportion of *bona fide* macrophages. SigF^+^ AM-like cells from HDM-treated *Tgfb1*^ΔCD11c^ mice also exhibited decreased expression of the homeostatic AM marker CD169^30^ compared with that exhibited by their littermate controls, but increased expression of programmed cell death 1 ligand 2 (PD-L2), which is associated with the inflammatory monocyte origin of macrophages during IL-4–dependent T2 immunity^31^ (Fig 3, *H* and see Fig E2, *E* and *F*). In contrast, expression of these proteins was comparable on SigF^–^ MPs from HDM-treated *Tgfb1*^ΔCD11c^ and *Tgfb1*^fl/fl^ mice and was much lower on Ly6C^hi^ monocytes (Fig 3, *H* and see Fig E2, *E*).

Both SigF^+^ AM-like cells and SigF^–^ MPs from *Tgfb1*^ΔCD11c^ mice, but not Ly6C^hi^ monocytes, expressed readily detectable mRNA for the classically activated macrophage marker gene *Nos2*/iNOS and high levels of the alternatively activated macrophage marker gene *Chil3* (see Fig E2, *G*). *Nos2* expression was increased in SigF^+^ AM-like cells from the *Tgfb1*^ΔCD11c^ mice as compared with in SigF^+^ AM-like cells from the *Tgfb1*^fl/fl^ controls, with a trend toward higher *Chil3* expression also observed (see Fig E2, *G*). Thus, the inflammatory CD11c^+^ MPs accumulating in HDM-treated *Tgfb1*^ΔCD11c^ mice display features of both classically and alternatively activated macrophages, as was previously observed with wild-type AMs from an adult AAD mouse model.^14^

Collectively, these results reveal a markedly dysregulated pulmonary MP response to early-life HDM inhalation in the context of TGF-β1 deficiency in CD11c^+^ cells.

### Inflammatory MP accumulation in HDM-treated *Tgfb1*^ΔCD11c^ mice is CCR2 dependent

**Fig 4:**
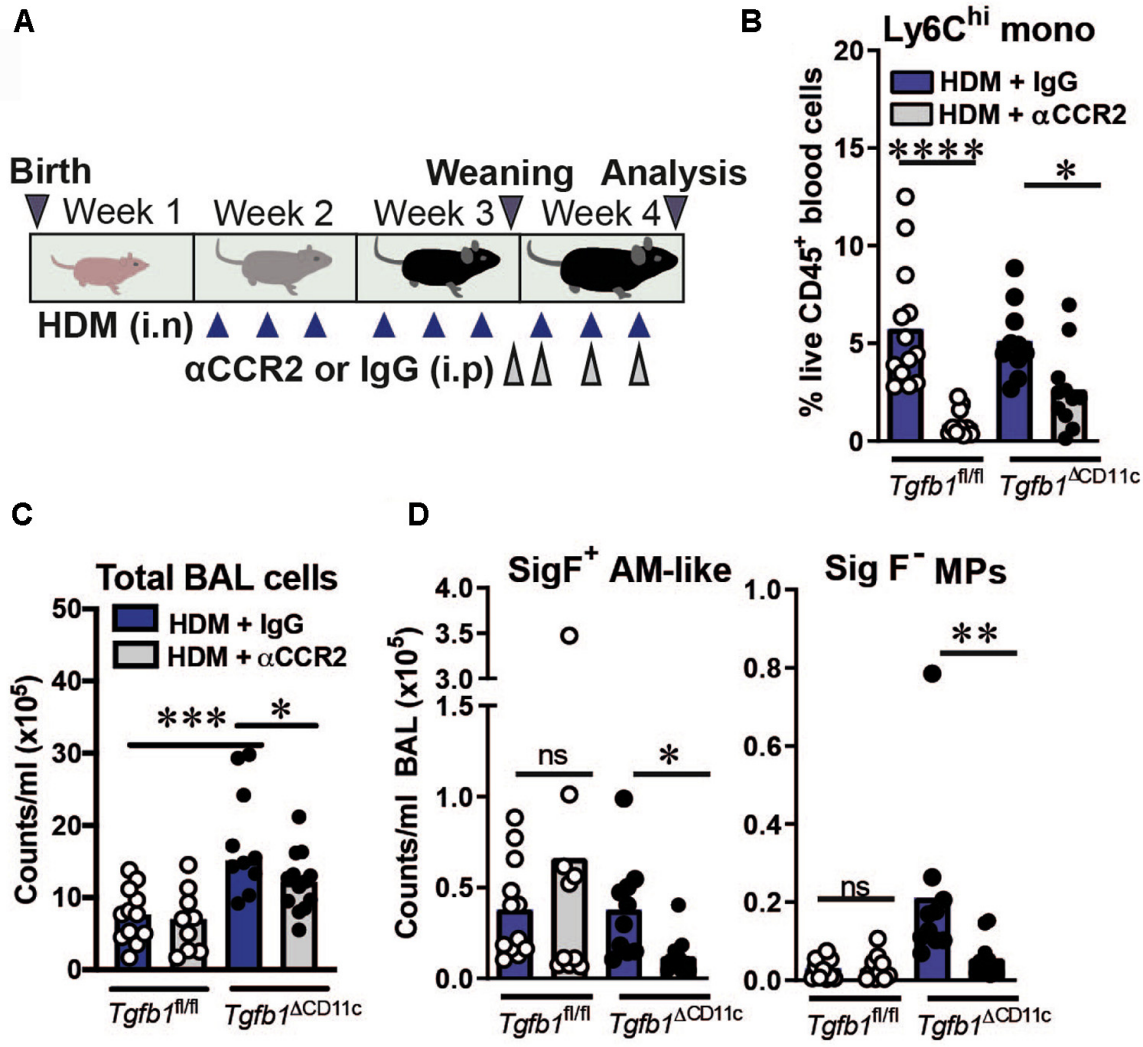
Inflammatory CD11c^+^ MP accumulation in HDM-treated *Tgfb1*^ΔCD11c^ mice is CCR2 dependent. **A,** CCR2 blocking (⍺CCR2) experiment schematic. **B,** Proportion of Ly6C^hi^ monocytes (Monos) in peripheral blood as determined by flow cytometry. **C,** Total cell counts in BAL fluid. **D,** Numbers of SigF^+^ AM-like cells and SigF^–^ MPs in BAL fluid, as determined by flow cytometry. Scatter plots and bar plots show data pooled from 2 individual experiments and means or medians with all individual replicates. Statistical results shown are from 1-way ANOVA with the Sidak *post hoc* test or Kruskal-Wallis test with the Dunn *post hoc* test. ∗*P* < .05; ∗∗*P* < .01; ∗∗∗*P* < .0001. *i.n*, Intranasal; i.p, intraperitoneal; *ns*, nonsignificant.

**Fig E3:**
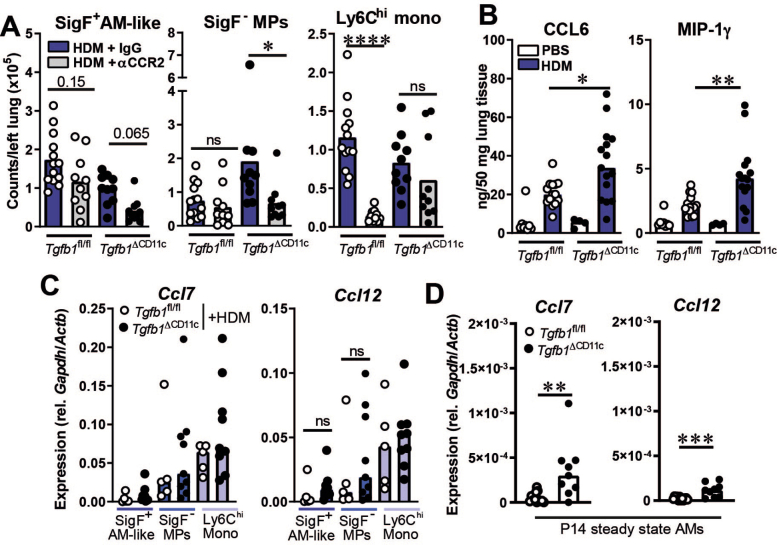


In contrast to the increase in total CD11c^+^CD64^+^F4/80^+^ inflammatory MPs in HDM-treated *Tgfb1*^ΔCD11c^ mice (Fig 3, *D* and see Fig E2, *A*), pulmonary Ly6C^hi^ monocyte numbers were not increased following HDM treatment (see Fig E2, *A*), suggesting that infiltrating Ly6C^hi^ monocytes in this model rapidly differentiate into inflammatory monocyte–derived cells. We therefore hypothesized that the inflammatory CD11c^+^ MPs that accumulate in the lungs of HDM-treated *Tgfb1*^ΔCD11c^ mice derive from recruited inflammatory monocytes. To test this hypothesis, we therapeutically administered an antibody to CCR2 (⍺CCR2),^27^ which is an essential receptor for inflammatory monocyte egress from bone marrow and mediator of their entry into tissues,^32^ to *Tgfb1*^ΔCD11c^ and *Tgfb1*^fl/fl^ mice throughout the final week of HDM exposures to systemically deplete Ly6C^hi^ monocytes (Fig 4, *A*). As expected, this ⍺CCR2 regimen substantially depleted circulating Ly6C^hi^ monocytes (Fig 4, *B*). Therapeutic ⍺CCR2 modestly reduced total airway cellularity but markedly decreased the numbers of SigF^+^ AM-like cells and SigF^–^ MPs in the BAL fluid and lung tissue of *Tgfb1*^ΔCD11c^, but not in *Tgfb1*^fl/fl^ control mice (Fig 4, *C* and *D* and see Fig E3, *A* in the Online Repository at www.jacionline.org). Neither population was completely ablated by anti-CCR2 (αCCR2) (Fig 4, *D* and see Fig E3, *A*), possibly owing to incomplete reduction of pulmonary Ly6C^hi^ monocyte numbers in *Tgfb1*^ΔCD11c^ mice (see Fig E3, *A*), which might allow CCR2-independent recruitment of residual circulating Ly6C^hi^ monocytes in αCCR2-treated *Tgfb1*^ΔCD11c^ mice. However, these results are consistent with the inflammatory CD11c^+^ MPs that accumulate following early-life HDM exposure in the context of TGF-β1 deficiency in CD11c^+^ cells requiring a substantial contribution from CCR2-dependent inflammatory monocytes.

### *Tgfb1*^ΔCD11c^ mice display increased monocyte chemokine production during allergic inflammation and at steady state

**Fig 5:**
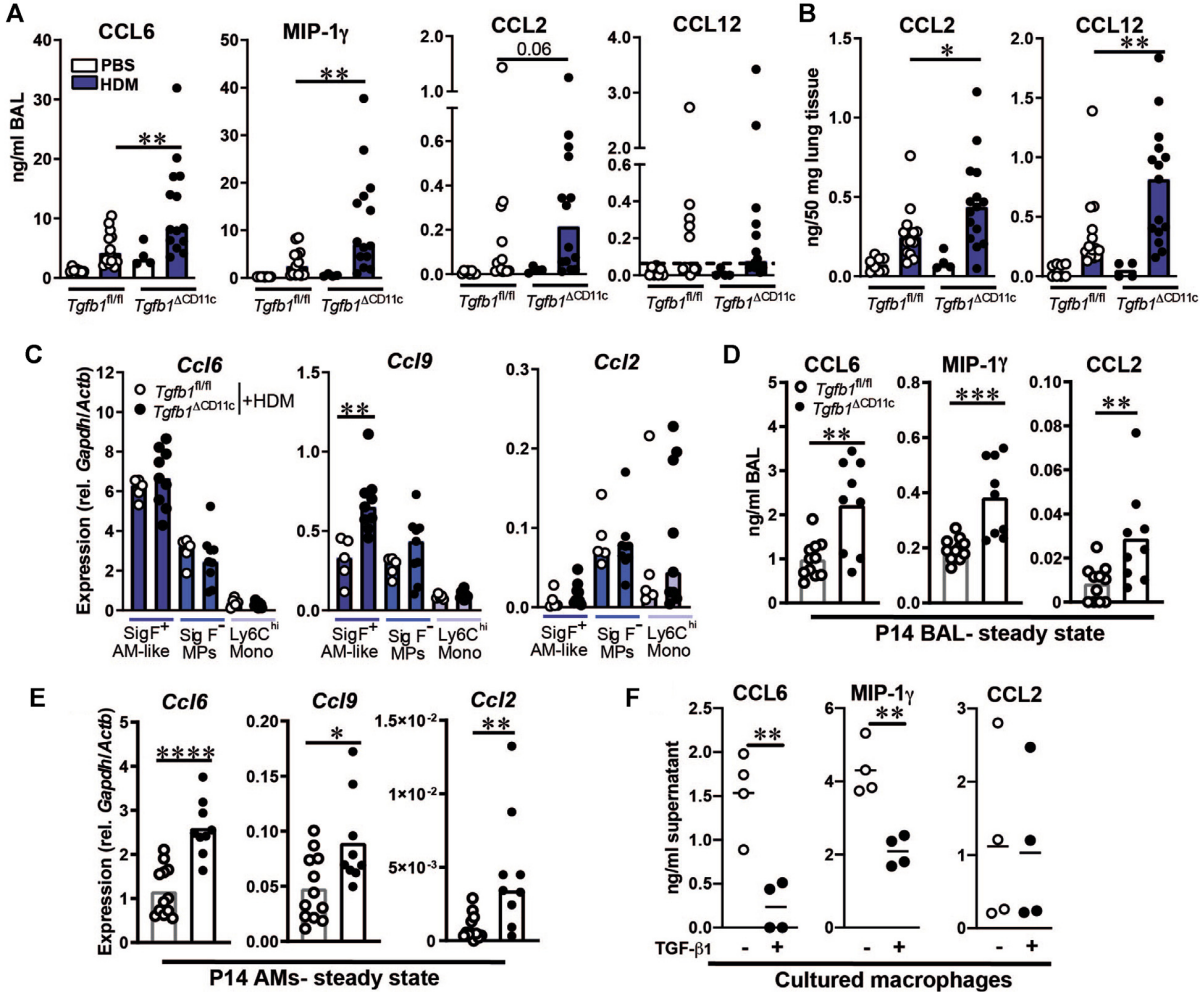
Dysregulated production of monocyte-attractant chemokines in *Tgfb1*^ΔCD11c^ mice. **A** and **B**, Concentrations of the indicated chemokines in BAL fluid (**A**) or lung tissue (**B**) as determined by ELISA. Dashed line indicates limit of detection. **C**, Relative quantitative PCR gene expression of the indicated chemokine genes in sorted MP populations in BAL fluid. **D** and **E**, BAL fluid was obtained from naive P14 mice and chemokine concentration measured in BAL fluid by ELISA (**D**), with gene expression determined in adhesion-purified AMs by quantitative PCR (**E**). **F**, Chemokine concentrations in supernatants of bone marrow–derived macrophages cultured with or without TGF-β1, as determined by ELISA. **A**, Results from a single assay using BAL fluid pooled from 3 mice per group. Scatter plots and bar plots (**A-E**) show means or medians and all individual replicates. **F**, Results from 1 of 2 experiments with similar results. Statistical results shown are from either Student unpaired *t* tests or Mann-Whitney *U* tests. ∗*P* < .05; ∗∗*P* < .01; ∗∗∗*P* < .001; ∗∗∗∗*P* < .0001. *Gapdh*, Glyceraldehyde-3-phosphate dehydrogenase; *Mono*, monocytes; *rel.*, relative to.

Having demonstrated dependency of inflammatory CD11c^+^ MP accumulation in HDM-treated *Tgfb1*^ΔCD11c^ mice on CCR2-dependent monocytes, we next sought evidence for dysregulated monocyte-attractant chemokine production in these mice. Because CCR1 and CCR2 are both required for maximal Ly6C^hi^ monocyte numbers in mouse lungs at the steady state^32^ and because both of these receptors can mediate monocyte accumulation during inflammation,^33^ we first quantified the ligands of these receptors in HDM-treated *Tgfb1*^ΔCD11c^ and *Tgfb1*^fl/fl^ control mice. The CCR1 agonists CCL6 and MIP-1γ (CCL9/10) were significantly more concentrated in the BAL fluid and lung tissue of HDM-treated *Tgfb1*^ΔCD11c^ mice than in *Tgfb1*^fl/fl^ controls (Fig 5, *A* and see Fig E3, *B*), whereas the CCR2 ligands CCL12 and CCL2 were more concentrated in lung tissue of *Tgfb1*^ΔCD11c^ mice, with a trend toward increased CCL2 level in the BAL fluid of knockouts (Fig 5, *A* and *B*). Notably, inflammatory CD11c^+^ MPs were identified as likely contributors to increased CCR1 ligand levels in *Tgfb1*^ΔCD11c^ mice. *Ccl6* and *Ccl9* genes were both highly expressed by SigF^+^ AM-like cells and SigF^–^ MPs from HDM-treated knockout and control mice, as compared with Ly6C^hi^ monocytes, with SigF^+^ AM-like cells from *Tgfb1*^ΔCD11c^ mice exhibiting higher *Ccl9* mRNA expression than in the littermate controls (Fig 5, *C*). Expression of the CCR2 ligands *Ccl2*, *Ccl7*, and *Ccl12* was relatively low in SigF^+^ AM-like cells (Fig 5, *C* and see Fig E3, *C*), whereas SigF^–^ MPs showed relatively high expression of *Ccl2* mRNA (Fig 5, *C*), suggesting that these cells contribute to elevated pulmonary CCL2 levels in HDM-treated *Tgfb1*^ΔCD11c^ mice, in which they are highly abundant (Fig 3, *D* and see Fig E2, *A*).

Thus, increased accumulation of inflammatory, largely CCR2-dependent, CD11c^+^ MPs following HDM treatment of neonatal mice with TGF-β1 deletion in CD11c^+^ cells is accompanied by increased pulmonary monocyte-attractant chemokine production.

Because accumulation of inflammatory monocyte–derived cells in the lung is thought to be an important early driver of HDM-elicited AAD,^10^^,^^22^ we hypothesized that increased inflammatory CD11c^+^ MP accumulation in HDM-treated *Tgfb1*^ΔCD11c^ mice would be associated with dysregulation of monocyte-attractant chemokine production during the early-life window of AM dysfunction in these mice, such that these mice are primed to overproduce CCR1 and CCR2 ligands before any allergen exposure. Correspondingly, in allergen-naive P14 mice, CCL6, MIP-1γ, and CCL2 concentrations were significantly increased in the BAL fluid of naive P14 *Tgfb1*^ΔCD11c^ mice compared with in the *Tgfb1*^fl/fl^ controls (Fig 5, *D*). CCL12 was not detectable in these samples (data not shown). The increased chemokine concentrations were at least partially due to dysregulated production by TGF-β1–deficient AMs, because *Ccl2*, *Ccl6*, and *Ccl9* gene expression was elevated in AMs from P14 *Tgfb1*^ΔCD11c^ mice (Fig 5, *E*). *Ccl7* and *Ccl12* gene expression was also higher in *Tgfb1*^ΔCD11c^ AMs than in *Tgfb1*^fl/fl^ controls (see Fig E3, *D*), but at much lower levels than *Ccl2*, *Ccl6*, and *Ccl9*. Thus, TGF-β1–deficient AMs are primed to produce monocyte-attractant chemokines in early life, before exposure to exogenous inflammatory stimuli, possibly reflecting a loss of autocrine regulation via TGF-β1.

Consistent with a role for direct TGF-β1–mediated suppression of constitutive chemokine expression by macrophages, exogenous TGF-β1 suppressed constitutive production of CCL6 and MIP-1γ, but not CCL2, by cultured mouse macrophages (Fig 5, *F*), presenting a mechanism by which autocrine TGF-β1 signaling may limit constitutive CCR1 ligand production by AMs in early life to maintain pulmonary immune homeostasis.

### *Tgfb1*^ΔCD11c^ mice display enhanced AAD following early-life HDM exposure

Recruited monocyte–derived cells are thought to act locally to promote T2 immune responses in the allergic lung.^10^^,^^22^ We therefore hypothesized that the increased inflammatory CD11c^+^ MP accumulation observed in *Tgfb1*^ΔCD11c^ mice exposed to HDM throughout early life would be accompanied by enhanced AAD.

**Fig 6:**
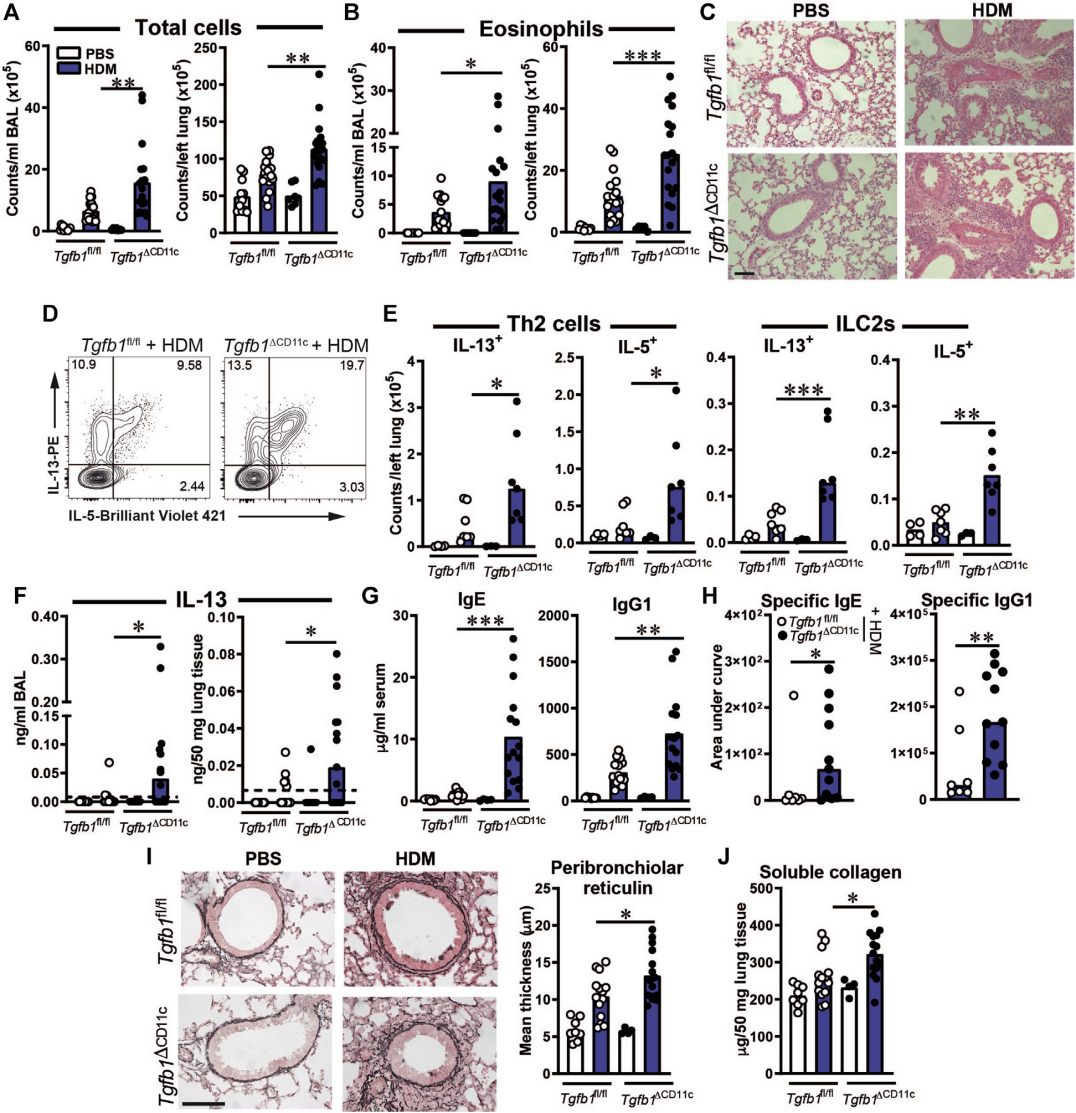
*Tgfb1*^ΔCD11c^ mice have more severe AAD after neonatal HDM exposure. **A**, Total BAL fluid and lung tissue cell counts. **B,** BAL fluid and lung eosinophil counts determined by flow cytometry. **C,** Representative images of hematoxylin and eosin–stained lung sections. Scale bar = 200 μm. **D,** Representative plots showing intracellular cytokine staining in lung CD4 T cells. **E**, Numbers of cytokine-positive lung CD4 T cells and ILCs. **F**, Concentrations of IL-13 in BAL fluid and lung tissue. Dashed lines show ELISA limit of detection. **G**, Concentrations of total IgE and IgG1 in serum. **H**, Area under curve analysis of HDM-specific IgE and IgG1 serum ELISA results. **I**, Representative images and quantification of peribronchiolar reticulin (*black fibers around airways*). Scale bar = 75 μm. **J**, Concentration of soluble collagen in lungs. Scatter plots and bar plots show means or medians and individual replicate values and are pooled from 2 to 4 experiments, except for (**E**), which shows 1 of 2 representative experiments. Statistical results shown are from either unpaired Student *t* tests or Mann-Whitney *U* tests. ∗*P* < .05; ∗∗*P* < .01; and ∗∗∗*P* < .001.

**Fig E4:**
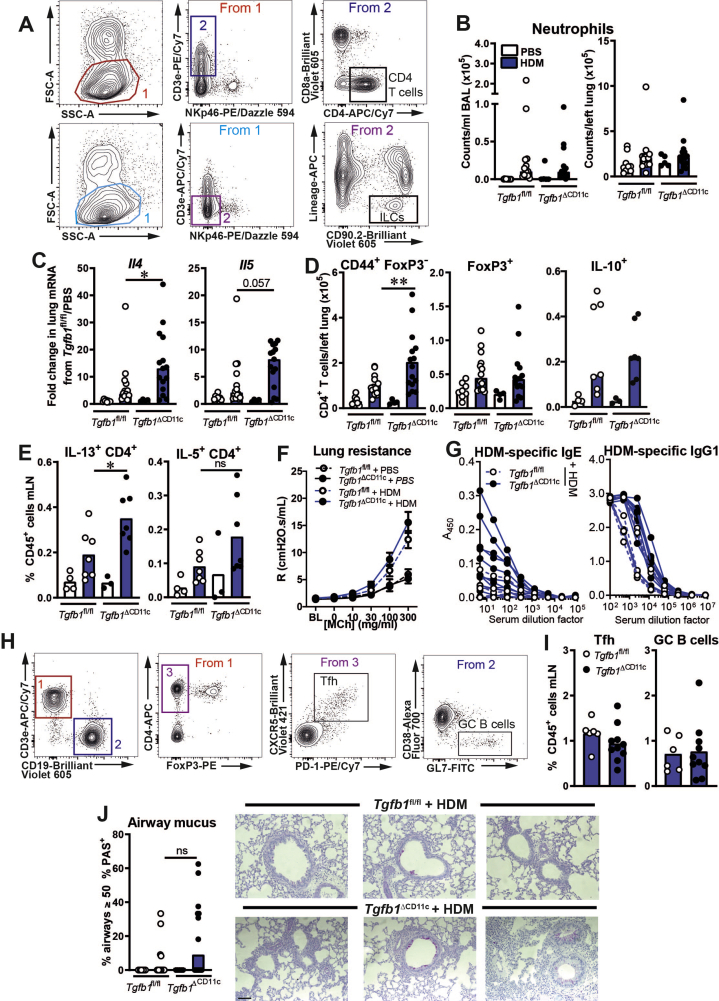


Notably, the predominantly C57BL/6 background strain of the *Tgfb1*^fl/fl^ mice makes them relatively refractory to HDM-driven AAD compared with the more allergy-prone BALB/c strain. Consequently, HDM treatment induced allergic pulmonary inflammation in *Tgfb1*^fl/fl^ mice, as determined by increased total leukocyte and eosinophil counts in the BAL fluid and lung tissue and peribronchiolar inflammation as visible on hematoxylin and eosin stained–sections (Fig 6, *A*-*C*), but these mice showed a minimal increase in numbers of IL-13– or IL-5–producing T_H_2 cells and group 2 innate lymphoid cells (ILC2s) in lung tissue and little to no detectable IL-13 in BAL fluid or lung homogenates (Fig 6, *D* and *F* and see Fig E4, *A* in the Online Repository at www.jacionline.org). BAL fluid and lung total cell and eosinophil counts were significantly elevated in HDM-treated *Tgfb1*^ΔCD11c^ mice compared with their littermate controls (Fig 6, *A* and *B*), as were with T_H_2 and ILC2 numbers and IL-13 concentrations (Fig 6, *D-F*), indicating that TGF-β1 deficiency in CD11c^+^ cells lowers the threshold for mounting a substantial T2 immune response to neonatal HDM exposure in these mice. HDM did not induce substantial neutrophilia in either knockout or control animals (see Fig E4, *B*). Lung CD4 T cells producing IFN-γ or IL-17A were several-fold less abundant than T_H_2 cells after HDM treatment and their numbers did not differ between the *Tgfb1*^ΔCD11c^ and control mice (data not shown).

The increased T_H_2 cell and ILC2 numbers in the lung were accompanied by increased *Il4* and *Il5* gene expression in the lung tissue (see Fig E4, *C*). The enhanced effector CD4 T-cell response in HDM-treated *Tgfb1*^ΔCD11c^ mice was confirmed in the absence of *ex vivo* stimulation for cytokine production by showing increased numbers of antigen-experienced CD44^+^FoxP3^–^ CD4 T cells, whereas the numbers of FoxP3^+^ regulatory T cells and CD4 T cells producing IL-10 were comparable in *Tgfb1*^ΔCD11c^ and control mice (see Fig E4, *D*), suggesting that deficiency in regulatory T*-*cell populations was not responsible for the augmented T2 lymphocyte response. IL-13^+^ CD4 T cells were also more abundant in lung-draining mediastinal lymph nodes (mLNs) of HDM-treated *Tgfb1*^ΔCD11c^ mice (see Fig E4, *E*).

Although HDM treatment of both *Tgfb1*^ΔCD11c^ and *Tgfb1*^fl/fl^ resulted in airway hyperresponsiveness to inhaled methacholine relative to PBS-treated controls, the heightened T2 immune response in HDM-treated *Tgfb1*^ΔCD11c^ mice was not accompanied by further increased airway hyperresponsiveness (see Fig E4, *F*). However, the concentrations of circulating total and HDM-specific IgE and IgG1 were markedly enhanced in HDM-treated *Tgfb1*^ΔCD11c^ mice (Fig 6, *G* and *H* and see Fig E4, *G*). Comparable mLN germinal center B-cell and T follicular helper cell frequencies were observed in HDM-treated *Tgfb1*^ΔCD11c^ and *Tgfb1*^fl/fl^ mice (see Fig E4, *H* and *I*), suggesting that the increased immunoglobulin titers resulted from the overall increased magnitude of T2 immunity rather than from specific germinal center dysregulation.

Concomitant with increased T2 immunity, subepithelial peribronchiolar reticulin thickness akin to the early reticular basement membrane remodeling, which was previously observed in preschool wheeze and difficult asthma in school-aged children,^34^, ^35^, ^36^ was increased more substantially by HDM treatment in *Tgfb1*^ΔCD11c^ mice than in the *Tgfb1*^fl/fl^ controls (Fig 6, *I*), indicating more established airway wall remodeling in these mice. Accordingly, soluble, newly synthesized, collagen was also more abundant in the lungs of these mice (Fig 6, *J*). Although a greater proportion of *Tgfb1*^ΔCD11c^ mice than control mice displayed high levels of airway mucus staining following HDM treatment, this was inconsistent and therefore not statistically significant (see Fig E4, *J*).

Thus, inflammatory MP accumulation elicited by HDM exposure in the context of early-life TGF-β1 deficiency in CD11c^+^ cells is accompanied by enhanced pulmonary T2 immunity and early airway remodeling.

### Dysregulated production of the proallergic chemokine CCL8 in HDM-treated *Tgfb1*^ΔCD11c^ mice

**Fig E5:**
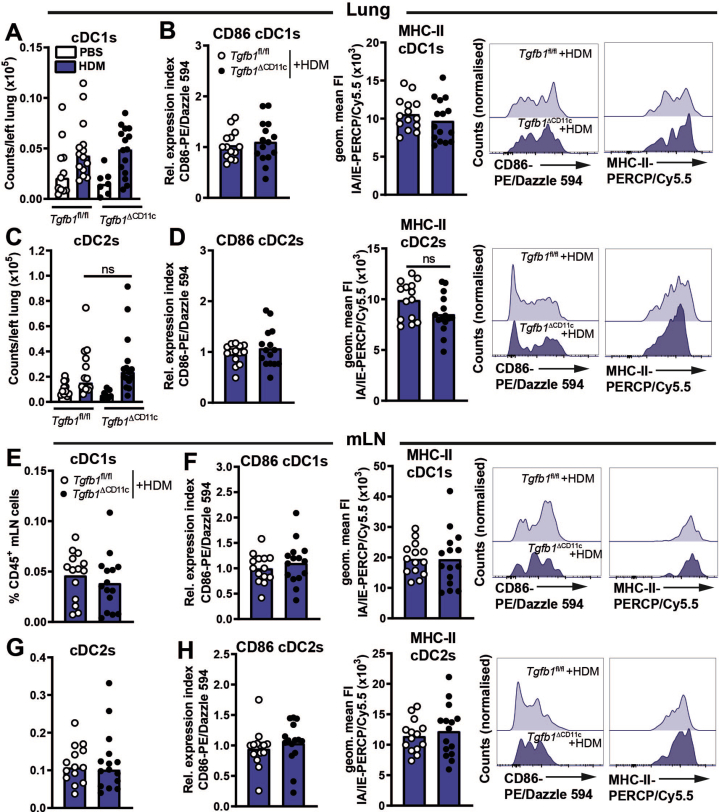


Given that pulmonary CD11c^+^ type 2 cDCs are crucial drivers of T2 immunity to HDM,^22^ we reasoned that the enhanced AAD in *Tgfb1*^ΔCD11c^ mice might be due to Cre-mediated dendritic cell–intrinsic *Tgfb1* deletion resulting in cDC dysfunction. However, type 1 cDC and type 2 cDC abundance and expression of the maturation markers CD86 and MHC class II were comparable in the lungs and mLNs of HDM-treated *Tgfb1*^ΔCD11c^ mice and *Tgfb1*^fl/fl^ control mice (see Fig E5 in the Online Repository at www.jacionline.org), thus presenting no evidence that pulmonary cDC number, maturation, or accumulation in mLNs was responsible for heightened HDM-driven AAD in *Tgfb1*^ΔCD11c^ mice.

**Fig E6:**
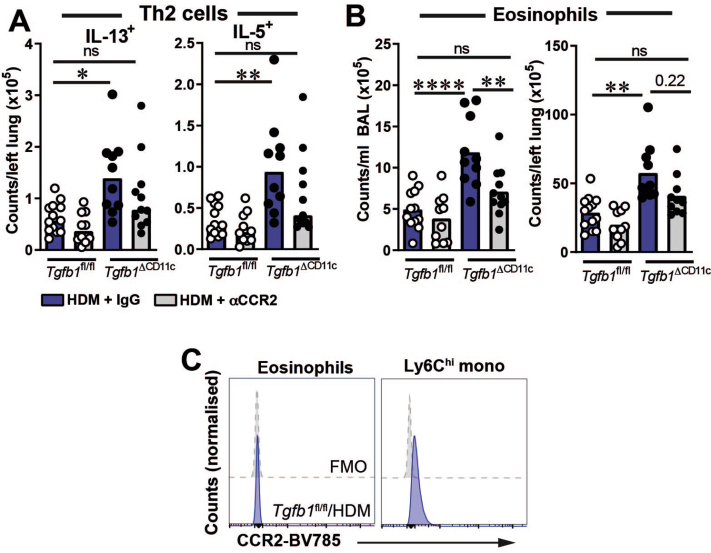


We next hypothesized that dysregulated SigF^+^ AM-like cells and SigF^–^ MPs were drivers of enhanced T2 immunity to HDM in *Tgfb1*^ΔCD11c^ mice. In support of a contribution of inflammatory CD11c^+^ MPs to heightened T2 immunity in these mice, αCCR2 treatment, which reduced the numbers of these cells in *Tgfb1*^ΔCD11c^ mice (Fig 4), also partially reduced the pulmonary T_H_2 cell and eosinophil numbers, such that the numbers were no longer significantly elevated above the levels in the *Tgfb1*^fl/fl^ controls and significantly diminished eosinophil counts in BAL fluid (see Fig E6, *A* and *B* in the Online Repository at www.jacionline.org). Reduced eosinophilia did not appear to be a direct effect of blockade of CCR2 signaling to eosinophils, because no CCR2 expression was detected on these cells during AAD (see Fig E6, *C*).

**Fig E7:**
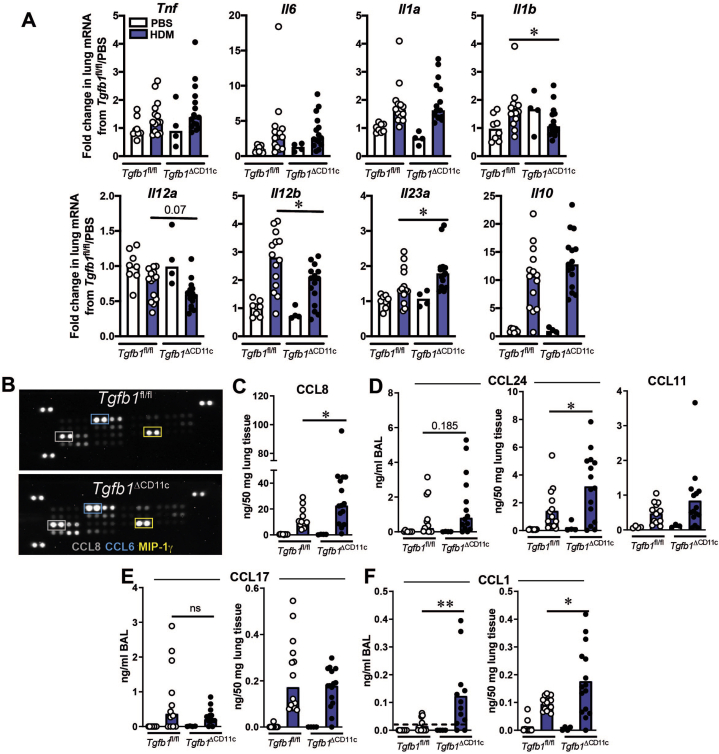


Reasoning that dysregulated inflammatory CD11c^+^ MPs in HDM-treated *Tgfb1*^ΔCD11c^ mice could facilitate HDM-driven AAD via increased local proinflammatory cytokine production, we examined expression of potentially MP-derived cytokine genes in lung tissue. Of these genes, only *Il23a*, encoding the α-subunit of IL-23, was more highly expressed in the lungs of *Tgfb1*^ΔCD11c^ mice than in the lungs of their littermate controls after HDM treatment (see Fig E7, *A*). However, expression of *Il12b*, encoding the second subunit of both IL-23 and IL-12, was decreased in HDM-treated *Tgfb1*^ΔCD11c^ mice (see Fig E7, *A*), indicating that there was not an overall skewing toward IL-23 production in these mice. Expression of *Il1b* was also decreased in *Tgfb1*^ΔCD11c^ mice compared with in their *Tgfb1*^fl/fl^ littermates after HDM treatment, with a trend toward decrease in *Il12a* expression (see Fig E7, *A*). These results thus do not support increased proinflammatory cytokine production as a correlate of the more severe AAD observed in *Tgfb1*^ΔCD11c^ mice exposed to HDM during early life.

**Fig 7:**
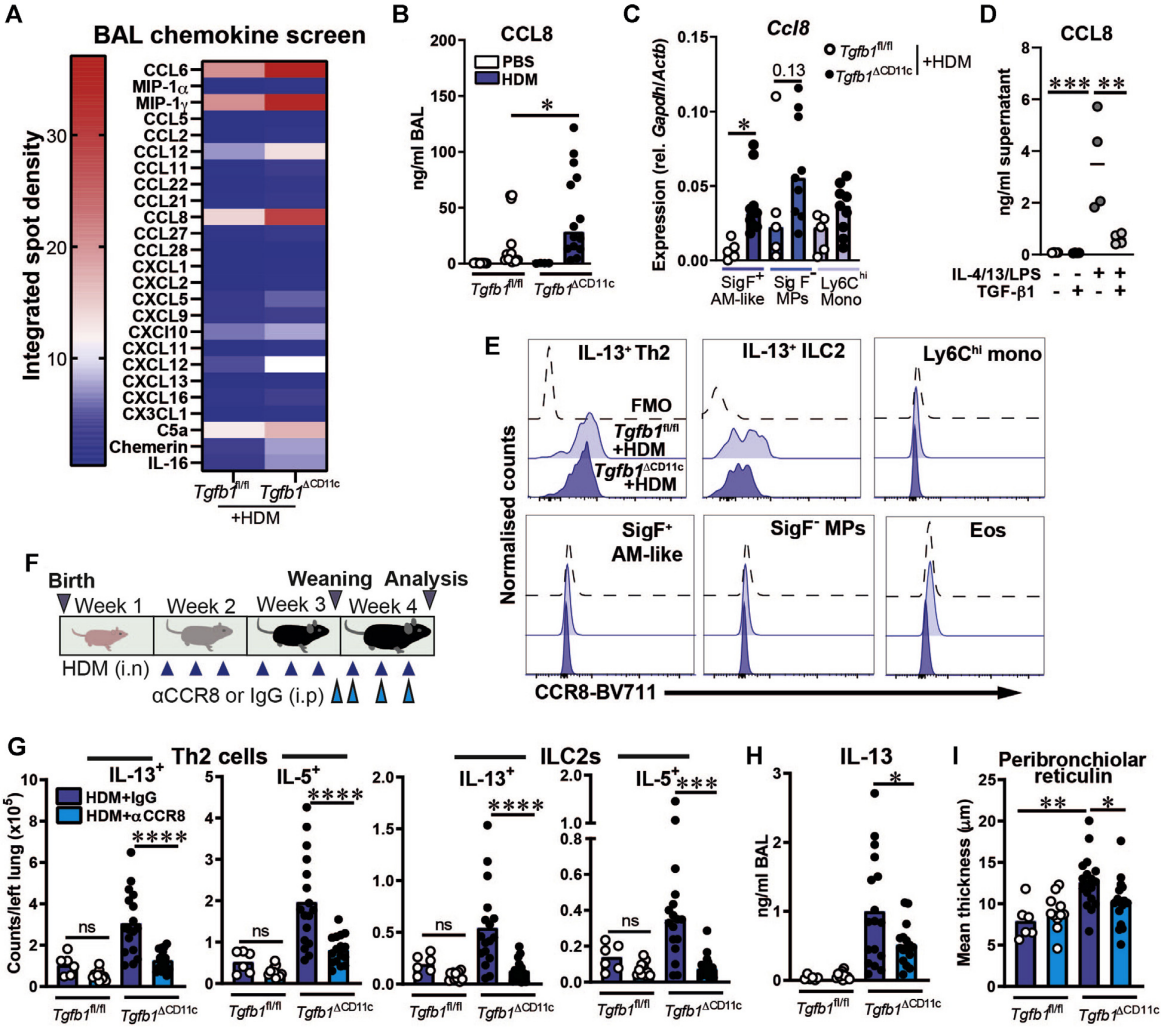
CCR8 signaling mediates enhanced inflammatory phenotypes in HDM-treated *Tgfb1*^ΔCD11c^ mice. **A**, Quantification of chemokine immunoblot on BAL fluid of HDM-treated mice. **B**, CCL8 concentration in BAL fluid measured by ELISA. **C,** Relative quantitative PCR gene expression of *Ccl8* in sorted MP populations in BAL fluid. **D,** CCL8 concentrations in supernatants of bone marrow–derived macrophages cultured plus or minus TGF-β1 and IL-4, IL-13 and LPS. **E,** Representative plots showing CCR8 surface staining compared with fluorescence minus one (FMO) controls. Plots are representative of 13 *Tgfb1*^fl/fl^ and 9 *Tgfb1*^ΔCD11c^ mice from 2 experiments. **F,** CCR8 blocking (⍺CCR8) experiment schematic. **G,** Numbers of IL-13–positive and IL-5–positive CD4 T cells and ILCs in lung tissue. **H,** IL-13 concentrations in BAL. **I,** Quantification of peribronchiolar reticulin. **A,** Results from a single assay pooling BAL fluid from 3 mice per group. Data in (**D**) are from 1 of 2 experiments with similar results. The remaining data are pooled from 2 experiments. Plots show means or medians and individual replicates. Statistical results shown are from 1-way ANOVA with the Sidak *post hoc* test for multiple comparisons and either Student unpaired *t* tests or Mann-Whitney *U* tests for single comparisons. ∗*P* < .05; ∗∗*P* < .01; ∗∗∗*P* < .001; ∗∗∗∗*P* < .0001. *Eos*, Eosinophils; *i.n*, intranasal; *i.p*, intraperitoneal; *Mono*, monocytes; *ns*, nonsignificant; *rel*., relative to.

Because both AMs and Siglec F^–^CD11c^+^MHC class II^+^CD64^+^ inflammatory MPs, which are likely to encompass some of the SigF^–^ MPs observed in our model, produce proallergic chemokines during HDM-driven AAD,^14^^,^^15^^,^^22^ we proposed that inflammatory CD11c^+^ MPs could promote an elevated level of T2 immunity in HDM-treated *Tgfb1*^ΔCD11c^ mice by chemokine production. To identify elevated levels of proallergic chemokines in the airways of our knockout mice, we screened the BAL fluid of HDM-treated *Tgfb1*^ΔCD11c^ and control mice by using a commercially available immunoblot array. This approach not only verified the previously observed increases in airway CCL6 and MIP-1γ concentrations in *Tgfb1*^ΔCD11c^ mice (Fig 5, *A*) but also highlighted a marked increase in CCL8 concentrations in BAL fluid, which was confirmed by ELISA in both in BAL fluid and lung tissue (Fig 7, *A* and *B* and see Fig E7, *B* and *C*).

Among other proallergic chemokines, the eosinophil chemoattractant CCL24/eotaxin 2 was also more concentrated in the lung tissue of HDM-treated *Tgfb1*^ΔCD11c^ mice than in that of *Tgfb1*^fl/fl^ mice, whereas CCL24 concentrations in the BAL fluid were highly variable but with a trend toward increase in knockouts (see Fig E7, *D*), suggesting that this chemokine might contribute to the elevated eosinophilia observed in these mice. In contrast, only a weak BAL fluid CCL11/eotaxin signal was detectable by chemokine array (Fig 7, *A*), and lung CCL11 concentrations did not differ between HDM-treated *Tgfb1*^ΔCD11c^ and control mice (see Fig E7, *D*). Concentrations of the T_H_2 cell chemokine CCL17 did not significantly differ in BAL fluid or lung tissue of *Tgfb1*^ΔCD11c^ and *Tgfb1*^fl/fl^ mice after HDM treatment (see Fig E7, *E*). CCL1, which shares the receptor CCR8 with CCL8 and can promote ILC2 proliferation and effector function,^37^ was more concentrated in the BAL fluid and lung tissue of HDM-treated *Tgfb1*^ΔCD11c^ mice than in the controls, but at around 100-fold lower levels than CCL8 (see Fig E7, *F*), indicating that CCL8 is the most abundant CCR8 ligand in this context.

Because we have previously demonstrated increased CCL8 expression by AMs during allergic airway inflammation driven by HDM or IL-33^14^^,^^15^^,^^38^ and others have also reported high relative CCL8 expression by CD11c^+^SigF^–^CD64^+^ inflammatory MPs that accumulate in HDM-treated mice,^22^ we postulated that the inflammatory CD11c^+^ MPs that accumulate in HDM-treated *Tgfb1*^ΔCD11c^ mice were a source of the elevated CCL8. Accordingly, *Ccl8* gene expression was detected in SigF^+^ AM-like cells, SigF^–^ MPs, and Ly6C^hi^ monocytes from HDM-treated mice and showed significantly greater expression in SigF^+^ AM-like cells from *Tgfb1*^ΔCD11c^ mice than in those from the controls (Fig 7, *C*).

No *Ccl8* gene expression was detected in steady-state AMs from P14 *Tgfb1*^ΔCD11c^ or *Tgfb1*^fl/fl^ mice (data not shown), suggesting that unlike CCL6 and MIP-1γ, TGF-β1–deficient AMs do not constitutively overproduce CCL8 in the steady state and that elevated CCL8 production by TGF-β1–deficient MPs may be reliant on additional inflammatory signals in the context of AAD. Accordingly, cultured mouse macrophages did not produce CCL8 with or without exogenous TGF-β1 in the absence of inflammatory stimuli. However, when activated with a moderate dose of LPS, plus IL-4 and IL-13, to mimic the inflammatory allergic airway environment, exogenous TGF-β1 restricted CCL8 production (Fig 7, *D*).

### The CCL8 receptor CCR8 is required for enhanced T2 lymphoid cell and airway remodeling responses to HDM in *Tgfb1*^ΔCD11c^ mice

**Fig E8:**
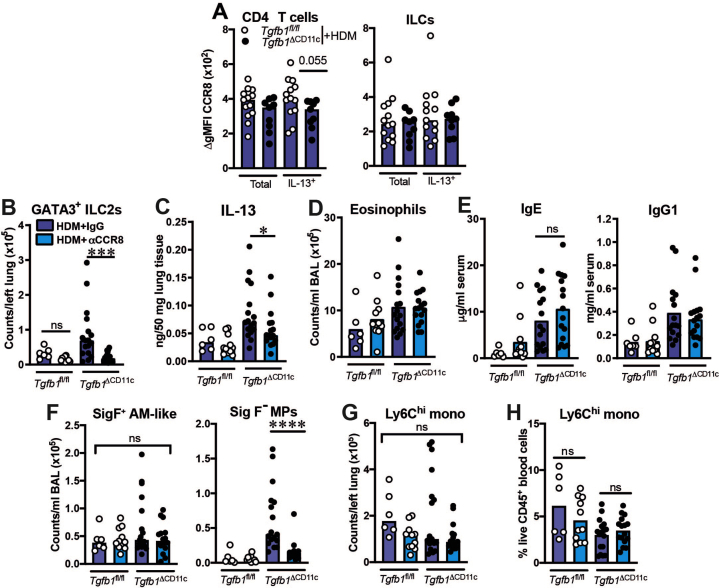


Because CCL8 can drive T2 lymphoid cell responses via its receptor CCR8,^38^^,^^39^ we reasoned that the elevated CCL8 production in *Tgfb1*^ΔCD11c^ mice treated with HDM during early life might drive augmented T2 immunity via a CCL8-CCR8 axis. CCR8 expression was detected on total and IL-13^+^ CD4 T cells and ILCs in HDM-treated *Tgfb1*^ΔCD11c^ and control mice, but not on MPs or eosinophils (Fig 7, *E* and see Fig E8, *A* in the Online Repository at www.jacionline.org), which is consistent with a role in T2 lymphoid cell function. Accordingly, anti-CCR8 (αCCR8) administration throughout the final week of HDM exposures (Fig 7, *F*) reduced pulmonary T_H_2 cell and ILC2 numbers in *Tgfb1*^ΔCD11c^ mice to near the *Tgfb1*^fl/fl^ control levels (Fig 7, *G*). The decreased IL-13^+^ ILC2 numbers with αCCR8 were not due solely to a reduction in IL-13 production because αCCR8 also reduced total GATA3^+^ CD90.2^+^Lin^–^ ILC2 numbers in the lungs of knockout mice (see Fig E8, *B*).

Therapeutic αCCR8 also significantly reduced BAL fluid and lung IL-13 concentrations and ameliorated the increased peribronchiolar reticulin thickness in HDM-treated *Tgfb1*^ΔCD11c^ mice (Fig 7, *H* and *I* and see Fig E8, *C*), suggesting that a pathogenic relationship exists between the CCL8-CCR8 axis, T2 lymphocytes, and airway remodeling in these mice. However, αCCR8 did not affect airway eosinophilia or circulating IgE and IgG1 concentrations (see Fig E8, *D* and *E*). SigF^+^ AM-like cell numbers in BAL fluid were unaffected by αCCR8 treatment, whereas SigF^–^ MP numbers in *Tgfb1*^ΔCD11c^ mice were significantly reduced (see Fig E7, *F*). However, because neither SigF^–^ MPs nor Ly6C^hi^ monocytes expressed CCR8 (Fig 7, *E*) and αCCR8 therapy did not affect pulmonary or circulating Ly6C^hi^ monocyte frequency (see Fig E8, *G* and *H*), the reduced Sig F^–^ MP numbers appeared to be downstream of the dampened T2 immune response following αCCR8 treatment.

Thus, *Tgfb1*^ΔCD11c^ mice produced elevated levels of pulmonary CCL8 following neonatal HDM exposure, derived in part from accumulating inflammatory CD11c^+^ MPs, and the CCL8 receptor CCR8 mediated heightened T2 immunity and airway wall remodeling in these mice.

## Discussion

The distinct immunologic environment of the neonatal lung influences responses to pathogens and aeroallergens,^3^^,^^40^, ^41^, ^42^ meaning that age is a major factor governing asthma susceptibility and phenotype.^4^^,^^5^ However, the mechanisms of development and regulation of the neonatal immune system in the face of initial exposures to pathogens and allergens are incompletely understood. The particular role of AMs in AAD remains unclear, with both protective regulatory and pathogenic proinflammatory and proremodeling roles proposed.^9^, ^10^, ^11^, ^12^, ^13^, ^14^ Here we have shown that TGF-β1 deficiency in CD11c^+^ MPs results in AM dysfunction in the crucial first weeks of life, precipitating dysregulated chemokine and inflammatory MP responses to inhaled HDM allergen that are concomitant with more severe allergic airway inflammation and remodeling.

We identified an age-specific effect of CD11c^+^ cell–restricted TGF-β1 deficiency on neonatal AM development, with surface marker and gene expression profiles suggestive of immature, dysfunctional AMs preceding the decrease in AM numbers in *Tgfb1*^ΔCD11c^ mice, which was previously reported at a single time point in late postnatal lung development at P28.^24^ Our results necessitate reinterpretation of the conclusions of Yu et al, who suggested that TGF-β1 action throughout AM development was entirely autocrine because mice with conditional TGF-β receptor knockout in AMs displayed reduced AM numbers as early as at P3.^24^ We have now demonstrated that TGF-β1 expression by AMs becomes necessary for maintenance of AM numbers only in late postnatal lung development, suggesting that partially compensatory TGF-β1 sources exist in the first weeks of life. Our longitudinal analysis therefore allowed us to study a window during the crucial first postnatal weeks in *Tgfb1*^ΔCD11c^ mice in which AMs were present but dysfunctional and incompletely matured, facilitating an augmented inflammatory response to HDM.

Chemokines function in a coordinated manner to drive allergic inflammation, with some acting upstream of others by recruiting additional chemokine-producing cells.^43^ AMs and inflammatory CD11c^+^Siglec F^–^CD64^+^ MPs have previously been described as local chemokine sources during allergic inflammation.^14^^,^^15^^,^^22^^,^^44^ We have demonstrated for the first time a role for TGF-β1 in specifically regulating the MP-derived chemokine repertoire in early life. *Tgfb1*^ΔCD11c^ mice displayed distinct patterns of increased CCR1, CCR2, and CCR8 ligand production at steady state and following neonatal HDM exposure. Moreover, we have demonstrated therapeutic effects of CCR2 and CCR8 blockade on components of the augmented immune response of *Tgfb1*^ΔCD11c^ mice to HDM, suggesting that agonists of these receptors act coordinately to lower the threshold for pathogenic immune responses to aeroallergen when TGF-β1–dependent AM maturation is impaired.

Expression of the major CCR1 ligands CCL6 and MIP-1γ and the major CCR2 ligand CCL2 was increased in TGF-β1–deficient neonatal AMs before any allergen exposure, suggesting that autocrine TGF-β1 signaling regulates expression of these chemokines in AMs during their postnatal development. Accordingly, we have also demonstrated suppression of constitutive *in vitro* CCL6 and MIP-1γ production from macrophages by exogenous TGF-β1. CCR2 and CCR1 are both required to achieve maximal pulmonary classical monocyte numbers at homeostasis,^32^ but they also mediate inflammatory monocyte recruitment to the lung.^33^ We have demonstrated CCR2-dependent accumulation of SigF^+^ AM-like cells, with increased expression of the inflammatory monocyte–derived macrophage markers CD11b and PD-L2 and SigF^–^ MPs (CD11c^+^CD64^+^F4/80^+^) in *Tgfb1*^ΔCD11c^ mice exposed to HDM over the first weeks of life, in which resident AMs display a dysfunctional phenotype.

The exact identity of the CCR2-dependent SigF^−^ MPs that accumulate in increased numbers in HDM-treated *Tgfb1*^ΔCD11c^ mice is unclear, with variable expression of the macrophage marker MerTK suggesting some heterogeneity within the population. Given the reduction in numbers of both inflammatory CD11c^+^ MP subsets in HDM-treated *Tgfb1*^ΔCD11c^ mice by CCR2 blockade, it can be speculated that the SigF^−^ MP population represents an intermediate state between inflammatory monocytes and CCR2-dependent SigF^+^ AM-like cells in these mice. However, independent CCR2-dependent origins of these populations also remain possible. It was clear that regardless of their precise origin and identity, CD11c^+^ MPs with inflammatory phenotypes accumulated in increased numbers following HDM exposure of *Tgfb1*^ΔCD11c^ mice. We therefore propose that increased classical monocyte-attractant chemokine expression by AMs in *Tgfb1*^ΔCD11c^ mice in early life, which might present a mechanism for gradual replacement of dysfunctional AMs by circulating monocytes at steady state, facilitates inflammatory CD11c^+^ MP accumulation in the context of additional signals elicited by HDM inhalation.

The mechanism linking the dysfunctional phenotype and increased chemokine expression of neonatal *Tgfb1*^ΔCD11c^ AMs to TGF-β1 deficiency remains to be determined. It is likely that key regulators of AM homeostatic function are disrupted in the absence of intrinsic TGF-β1 expression, contributing to their dysregulated phenotype. Indeed, we have shown reduced expression of the gene encoding the essential AM transcription factor PPAR-γ^28^ in TGF-β1–deficient AMs in early life. Another possible contributor is RhoA, a TGF-β–inducible molecule that was highly expressed in pediatric AMs in our study and has been implicated in macrophage polarization and function.^45^

CCL8, a key mediator of T_H_2 and ILC2 responses,^38^^,^^39^ showed dynamics of expression distinct from those of CCR1 and CCR2 ligands. CCL8 was not expressed by wild-type or TGF-β1–deficient neonatal AMs at steady state but was expressed by inflammatory SigF^+^ AM-like cells and SigF^–^ MPs during established AAD, accompanied by increased pulmonary CCL8 concentrations in HDM-treated *Tgfb1*^ΔCD11c^ mice, thus suggesting that increased CCL8 production occurs downstream of the initial dysregulated inflammatory MP response to HDM in these mice. Importantly, we have shown the CCL8 receptor CCR8 to be central to AAD pathogenesis in *Tgfb1*^ΔCD11c^ mice. *Tgfb1*^ΔCD11c^ mice displayed augmented T_H_2 and ILC2 responses to HDM, with increased pulmonary IL-13 concentrations and peribronchiolar reticulin deposition, which were reduced by therapeutic CCR8 blockade.

Whether the increased CCL8 production by SigF^+^ AM-like cells in HDM-treated *Tgfb1*^ΔCD11c^ mice is due to intrinsic TGF-β1 deficiency in these cells remains to be seen. Although our data showing TGF-β1 suppression of CCL8 production by *in vitro*–stimulated macrophages supports such a direct mechanism, it remains possible that the likely monocyte origin of the majority of SigF^+^ AM-like cells in these mice endows them with greater capacity for CCL8 production, as was recently demonstrated for IL-6 in monocyte-derived AMs during influenza infection.^19^ Both mechanisms could conceivably contribute to the enhanced CCL8 observed in our model. Regardless, we can conclude from our data that the dysregulated immune response to HDM in neonatal mice with TGF-β1 deficiency in CD11c^+^ cells results in production of high levels of CCL8 derived partially from the inflammatory MPs that accumulate in these mice, which drives pathogenic T2 lymphocyte and airway remodeling responses to HDM via CCR8.

A previous study of the tolerogenic function of AMs in mice implicated AM-derived TGF-β as a mediator promoting FoxP3 expression in naive CD4 T cells.^46^ However, this analysis of TGF-β function was performed by using an *in vitro* coculture system, and the extent to which such interactions between AMs and naive CD4 T cells occur during allergic sensitization *in vivo* is unclear. Notably, the augmented early-life AAD in *Tgfb1*^ΔCD11c^ mice in our present study was not accompanied by a failure to accumulate FoxP3^+^ CD4 T cells in lung tissue, suggesting that this mechanism was not occurring in our neonatal *in vivo* model.

Although our results in mice are complemented by RNA-Seq analysis supporting TGF-β1 as a key regulator of human AM gene expression in early life, it remains to be determined whether TGF-β1 signaling promotes maturation and restricts chemokine expression in these AMs to limit inflammatory responses to environmental stimuli and whether this is disrupted in childhood asthma. AM phenotype is governed by a combination of local tissue signals,^8^ so it is likely that several factors control AM phenotype in the complex human setting. Further investigation into the factors regulating macrophage maturity and phenotype in the pediatric airway and their relationship to susceptibility to early-life pulmonary inflammation is therefore warranted.

Early life presents not only a crucial period of vulnerability of the pulmonary immune system to infection and allergic sensitization but also a window of opportunity for therapeutic manipulation of immunity.^5^ Better understanding of the intrinsic and extrinsic regulators of the thresholds for initiation of protective and pathogenic immunity in the neonatal lung is therefore vital. We have identified a pivotal and previously unappreciated temporal role for TGF-β1–dependent AM maturation in controlling the magnitude of immunologic and pathologic responses to neonatal HDM exposure. Our findings highlight a combinatorial relationship between TGF-β1, AM maturation, the pulmonary chemokine repertoire, monocyte-derived cell recruitment, and T2 immunity that sets the threshold for mounting pathogenic allergic responses in early life.

**Key messages**

• CD11c^+^ MP–derived TGF-β1 regulates AM maturation and CCR2-dependent inflammatory MP responses to inhaled HDM allergen in early life.
• HDM-treated *Tgfb1*^ΔCD11c^ mice develop heightened AAD that is dependent on the CCL8 receptor CCR8.

## Supplementary data

Online Repository

## References

[1] Lee A.H., Shannon C.P., Amenyogbe N., Bennike T.B., Diray-Arce J., Idoko O.T. (2019). Dynamic molecular changes during the first week of human life follow a robust developmental trajectory. Nat Commun.

[2] Olin A., Henckel E., Chen Y., Lakshmikanth T., Pou C., Mikes J. (2018). Stereotypic immune system development in newborn children. Cell.

[3] Lambert L., Culley F.J. (2017). Innate immunity to respiratory infection in early life. Front Immunol.

[4] Lloyd C.M., Saglani S. (2017). Development of allergic immunity in early life. Immunol Rev.

[5] Lloyd C.M., Saglani S. (2019). Opening the window of immune opportunity: treating childhood asthma. Trends Immunol.

[6] Guilliams M., De Kleer I., Henri S., Post S., Vanhoutte L., De Prijck S. (2013). Alveolar macrophages develop from fetal monocytes that differentiate into long-lived cells in the first week of life via GM-CSF. J Exp Med.

[7] Byrne A.J., Mathie S.A., Gregory L.G., Lloyd C.M. (2015). Pulmonary macrophages: key players in the innate defence of the airways. Thorax.

[8] Hussell T., Bell T.J. (2014). Alveolar macrophages: plasticity in a tissue-specific context. Nat Rev Immunol.

[9] Mathie S.A., Dixon K.L., Walker S.A., Tyrrell V., Mondhe M., O'Donnell V.B. (2015). Alveolar macrophages are sentinels of murine pulmonary homeostasis following inhaled antigen challenge. Allergy.

[10] Zaslona Z., Przybranowski S., Wilke C., van Rooijen N., Teitz-Tennenbaum S., Osterholzer J.J. (2014). Resident alveolar macrophages suppress, whereas recruited monocytes promote, allergic lung inflammation in murine models of asthma. J Immunol.

[11] Draijer C., Robbe P., Boorsma C.E., Hylkema M.N., Melgert B.N. (2013). Characterization of macrophage phenotypes in three murine models of house-dust-mite-induced asthma. Mediators Inflamm.

[12] Byrne A.J., Weiss M., Mathie S.A., Walker S.A., Eames H.L., Saliba D. (2017). A critical role for IRF5 in regulating allergic airway inflammation. Mucosal Immunol.

[13] Staples K.J., Hinks T.S., Ward J.A., Gunn V., Smith C., Djukanovic R. (2012). Phenotypic characterization of lung macrophages in asthmatic patients: overexpression of CCL17. J Allergy Clin Immunol.

[14] Branchett W.J., O'Garra A., Lloyd C.M. (2020). Transcriptomic analysis reveals diverse gene expression changes in airway macrophages during experimental allergic airway disease. Wellcome Open Res.

[15] Branchett W.J., Stölting H., Oliver R.A., Walker S.A., Puttur F., Gregory L.G. (2020). A T cell–myeloid IL-10 axis regulates pathogenic IFN-γ–dependent immunity in a mouse model of type 2–low asthma. J Allergy Clin Immunol.

[16] Hashimoto D., Chow A., Noizat C., Teo P., Beasley M.B., Leboeuf M. (2013). Tissue-resident macrophages self-maintain locally throughout adult life with minimal contribution from circulating monocytes. Immunity.

[17] Misharin A.V., Morales-Nebreda L., Reyfman P.A., Cuda C.M., Walter J.M., McQuattie-Pimentel A.C. (2017). Monocyte-derived alveolar macrophages drive lung fibrosis and persist in the lung over the life span. J Exp Med.

[18] Lee Y.G., Jeong J.J., Nyenhuis S., Berdyshev E., Chung S., Ranjan R. (2015). Recruited alveolar macrophages, in response to airway epithelial-derived monocyte chemoattractant protein 1/CCl2, regulate airway inflammation and remodeling in allergic asthma. Am J Respir Cell Mol Biol.

[19] Aegerter H., Kulikauskaite J., Crotta S., Patel H., Kelly G., Hessel E.M. (2020). Influenza-induced monocyte-derived alveolar macrophages confer prolonged antibacterial protection. Nat Immunol.

[20] Byrne A.J., Powell J.E., O’Sullivan B.J., Ogger P.P., Hoffland A., Cook J. (2020). Dynamics of human monocytes and airway macrophages during healthy aging and after transplant. J Exp Med.

[21] Liu Z., Gu Y., Chakarov S., Bleriot C., Kwok I., Chen X. (2019). Fate mapping via Ms4a3-expression history traces monocyte-derived cells. Cell.

[22] Plantinga M., Guilliams M., Vanheerswynghels M., Deswarte K., Branco-Madeira F., Toussaint W. (2013). Conventional and monocyte-derived CD11b(+) dendritic cells initiate and maintain T helper 2 cell-mediated immunity to house dust mite allergen. Immunity.

[23] Batlle E., Massague J. (2019). Transforming growth factor-beta signaling in immunity and cancer. Immunity.

[24] Yu X., Buttgereit A., Lelios I., Utz S.G., Cansever D., Becher B. (2017). The cytokine TGF-beta promotes the development and homeostasis of alveolar macrophages. Immunity.

[25] Caton M.L., Smith-Raska M.R., Reizis B. (2007). Notch-RBP-J signaling controls the homeostasis of CD8- dendritic cells in the spleen. J Exp Med.

[26] Azhar M., Yin M., Bommireddy R., Duffy J.J., Yang J., Pawlowski S.A. (2009). Generation of mice with a conditional allele for transforming growth factor beta 1 gene. Genesis.

[27] Mack M., Cihak J., Simonis C., Luckow B., Proudfoot A.E., Plachy J. (2001). Expression and characterization of the chemokine receptors CCR2 and CCR5 in mice. J Immunol.

[28] Schneider C., Nobs S.P., Kurrer M., Rehrauer H., Thiele C., Kopf M. (2014). Induction of the nuclear receptor PPAR-gamma by the cytokine GM-CSF is critical for the differentiation of fetal monocytes into alveolar macrophages. Nat Immunol.

[29] Duan M., Steinfort D.P., Smallwood D., Hew M., Chen W., Ernst M. (2016). CD11b immunophenotyping identifies inflammatory profiles in the mouse and human lungs. Mucosal Immunol.

[30] Ducreux J., Crocker P.R., Vanbever R. (2009). Analysis of sialoadhesin expression on mouse alveolar macrophages. Immunol Lett.

[31] Gundra U.M., Girgis N.M., Ruckerl D., Jenkins S., Ward L.N., Kurtz Z.D. (2014). Alternatively activated macrophages derived from monocytes and tissue macrophages are phenotypically and functionally distinct. Blood.

[32] Dyer D.P., Medina-Ruiz L., Bartolini R., Schuette F., Hughes C.E., Pallas K. (2019). Chemokine receptor redundancy and specificity are context dependent. Immunity.

[33] Shi C., Pamer E.G. (2011). Monocyte recruitment during infection and inflammation. Nat Rev Immunol.

[34] Saglani S., Payne D.N., Zhu J., Wang Z., Nicholson A.G., Bush A. (2007). Early detection of airway wall remodeling and eosinophilic inflammation in preschool wheezers. Am.J.Respir.Crit Care Med.

[35] Saglani S., Mathie S.A., Gregory L.G., Bell M.J., Bush A., Lloyd C.M. (2009). Pathophysiological features of asthma develop in parallel in house dust mite-exposed neonatal mice. Am J Respir Cell Mol Biol.

[36] Bossley C.J., Fleming L., Gupta A., Regamey N., Frith J., Oates T. (2012). Pediatric severe asthma is characterized by eosinophilia and remodeling without T_H_2 cytokines. J Allergy Clin Immunol.

[37] Knipfer L., Schulz-Kuhnt A., Kindermann M., Greif V., Symowski C., Voehringer D. (2019). A CCL1/CCR8-dependent feed-forward mechanism drives ILC2 functions in type 2-mediated inflammation. J Exp Med.

[38] Puttur F., Denney L., Gregory L.G., Vuononvirta J., Oliver R., Entwistle L.J. (2019). Pulmonary environmental cues drive group 2 innate lymphoid cell dynamics in mice and humans. Sci Immunol.

[39] Islam S.A., Chang D.S., Colvin R.A., Byrne M.H., McCully M.L., Moser B. (2011). Mouse CCL8, a CCR8 agonist, promotes atopic dermatitis by recruiting IL-5+ T_H_2 cells. Nat Immunol.

[40] Bachus H., Kaur K., Papillion A.M., Marquez-Lago T.T., Yu Z., Ballesteros-Tato A. (2019). Impaired tumor-necrosis-factor-alpha-driven dendritic cell activation limits lipopolysaccharide-induced protection from allergic inflammation in infants. Immunity.

[41] Saglani S., Gregory L.G., Manghera A.K., Branchett W.J., Uwadiae F., Entwistle L.J. (2018). Inception of early-life allergen-induced airway hyperresponsiveness is reliant on IL-13(+)CD4(+) T cells. Sci Immunol.

[42] Saluzzo S., Gorki A.D., Rana B.M.J., Martins R., Scanlon S., Starkl P. (2017). First-breath-induced type 2 pathways shape the lung immune environment. Cell Rep.

[43] Gonzalo J.A., Lloyd C.M., Wen D., Albar J.P., Wells T.N., Proudfoot A. (1998). The coordinated action of CC chemokines in the lung orchestrates allergic inflammation and airway hyperresponsiveness. J Exp Med.

[44] Ma B., Zhu Z., Homer R.J., Gerard C., Strieter R., Elias J.A. (2004). The C10/CCL6 chemokine and CCR1 play critical roles in the pathogenesis of IL-13-Induced inflammation and remodeling. J Immunol.

[45] Zhang Y., Saradna A., Ratan R., Ke X., Tu W., Do D.C. (2020). RhoA/Rho-kinases in asthma: from pathogenesis to therapeutic targets. Clin Transl Immunol.

[46] Soroosh P., Doherty T.A., Duan W., Mehta A.K., Choi H., Adams Y.F. (2013). Lung-resident tissue macrophages generate Foxp3+ regulatory T cells and promote airway tolerance. J Exp Med.

