# Supplementary material for: Airway macrophage-intrinsic TGF-β1 regulates pulmonary immunity during early-life allergen exposure
Source: J Allergy Clin Immunol. 2021 May;147(5):1892–906. doi: 10.1016/j.jaci.2021.01.026 (PMC8098862; doi:10.1016/j.jaci.2021.01.026)
Supplement: Online Repository [file mmc1.docx]

**Supplementary Methods**

**Mouse experiment design**

Mouse experiments were performed a minimum of two times and data combined where possible, as indicated in figure legends. Experiments with bone-marrow-derived macrophages experiments were performed once with cells derived from male and once from female mice, with comparable results obtained from mice of both sexes. Experiments with *Tgfb1*^ΔCD11c^ mice were performed with roughly equal proportions of males and females, with no sex differences apparent in the parameters we examined. Minimal experimental group sizes were determined based on prior experience with the parameters measured and the final group sizes were dependent on the number of *Tgfb1*^ΔCD11c^ and *Tgfb1*^flfl^ littermate control mice born by random Mendelian inheritance. Group sizes are indicated in the figures and their respective legends. Individual litters were randomly assigned to treatment groups. Cell counting and histological analyses were performed while blinded to the experimental group.

**Lung function analysis**

Mice were anaesthetised with 50 mg/kg intraperitoneal sodium pentobarbital and 100 mg/kg intramuscular ketamine, tracheostomised and mechanically ventilated using the flexiVent small animal ventilator (Scireq) via 19G tracheal cannulas. Mice were ventilated and snapshot perturbation measurements taken in response to increasing concentrations of nebulised methacholine (Sigma-Aldrich) as previously described^1^.

**Mouse tissue processing**

Mice were euthanized by intraperitoneal overdose of pentobarbital and exsanguination by cardiac puncture. For serum collection, blood was transferred into serum separator tubes (Becton Dickinson) and centrifuged at 10 000 x *g* for five minutes to separate serum. To prepare blood leukocytes for flow cytometry, approximately 150 μl of blood was transferred to 1.5 ml microcentrifuge tubes containing 20 μl of 500 U/ml heparin (Sigma-Aldrich). Blood cells were then washed in two changes of ammonium chloride erythrocyte lysis buffer (0.155 M ammonium chloride, 10 mM potassium bicarbonate and 0.11 mM disodium EDTA in deionised water at pH 7.2-7.4), before suspending in complete RPMI (cRPMI; RPMI 1640 with L-glutamine and phenol red + 10 % FCS + 100 U/ml penicillin and streptomycin; all from Gibco, Thermo Fisher).

Bronchoalveolar lavage (BAL) was performed via 23 G and 19 G tracheal cannulas for two and four week old mice, respectively. Three different BAL protocols were used. For analysis of two week old mice, airways were flushed three times with 0.2 ml of PBS, before pooling fractions, centrifuging to pellet cells, storing supernatant at -80 ^o^C and suspending pellets in cRPMI. In some experiments, AMs were adhesion-purified from BAL by adhering BAL suspensions to 48 well tissue culture plates in serum-free advanced RPMI, supplemented with 100 U/ml penicillin and streptomycin and 2mM L-glutamine (adRPMI; all Gibco, Thermo Fisher), for two hours and cell layers washed with PBS before lysis for RNA extraction. Standard BAL analysis of four week old mice was performed as above, but using three 0.4 ml volumes of PBS. For high yield BAL for cell sorting from four week old mice, airways were lavaged a total of seven times with 0.8 ml of 5 mM EDTA (Invitrogen, Thermo Fisher) in PBS, leaving the fourth volume of EDTA in the lungs for five minutes to help release cells adherent to the epithelium.

Mediastinal lymph nodes were dissociated through 70 μm cell strainers and suspended in cRPMI. Lungs were dissected into individual lobes. Right superior lobes were inflated via a major airway with PBS and fixed for 24 hours in 10 % buffered neutral formalin for histological analysis. Right middle lobes were flash frozen in liquid nitrogen and homogenised at 50 mg tissue/ml in Hank’s Buffered Salt Saline (Gibco, Thermo Fisher) in the presence of the cOmplete protease inhibitor cocktail (one tablet per 50 ml buffer, Roche), using a FastPrep 24 bead homogeniser and Lysing Matrix D (MP Biomedicals). Homogenates were centrifuged at 11 000 x *g* for 10 minutes at 4 ^o^C to pellet debris and supernatants stored at -80 ^o^C until the time of protein quantification. Right post-caval lobes were immersed in RNALater reagent (Sigma-Aldrich) for 24 hours at 4 ^o^C then frozen at -80 ^o^C for subsequent RNA extraction. Whole left lungs were minced with scissors before digesting for one hour at 37 ^o^C in cRPMI containing 150 μg/ml collagenase D and 25 μg/ml DNase I (both Roche) in a shaking water bath. Digested lung tissue was dissociated through 70 μm cell strainers, before incubating for five minutes in ammonium chloride erythrocyte lysis buffer and suspending in cRPMI. Total BAL and lung cell counts were performed by staining cells with 0.02 % crystal violet (Sigma-Aldrich) and 700 mM acetic acid solution in PBS and counting manually on haemocytometer slides.

**Bone marrow isolation, macrophage differentiation and *in vitro* TGF-β1 response studies**

Femurs were flushed with cRPMI using 26 G needles and bone marrow cells incubated for five minutes with ammonium chloride erythrocyte lysis buffer before culturing 3 x 10^6^ cells in a volume of 10 ml cRPMI, supplemented with 55 uM tissue culture-grade β-mercaptoethanol (Gibco, Thermo Fisher) and 100 ng/ml human M-CSF (PeproTech) in 10 cm petri dishes. On day three of culture, half of the media volume was replaced by fresh cRPMI with β-mercaptoethanol and M-CSF. On day eight, plates were washed with PBS and adherent differentiated macrophages removed by brief incubation with 0.05 % Trypsin-EDTA (Gibco, Thermo Fisher). 10^5^ cells per well were then adhered to wells of 48 well plates in serum-free adRPMI. Cells were incubated with either adRPMI alone or supplemented with 20 ng/ml murine TGF-β1 with carrier protein (R&D Systems, prepared in acidic solution as per manufacturer’s recommendations) for 30 minutes prior to spiking in either combined 10 ng/ml murine IL-4 (PeproTech), 10 ng/ml murine IL-13 (PeproTech) and 10 ng/ml Lipopolysaccharides from *E. coli* 055:B5 (BioXtra, Sigma-Aldrich) or adRPMI control for the remainder of culture duration. Medium was harvested after 15 hours, centrifuged to remove any non-adherent cells and supernatants frozen at -80 ^o^C until analysis.

**Histological staining and analysis**

Formalin-fixed lung lobes were wax-embedded and 4 μm sections prepared and mounted on glass microscope slides. Periodic Acid-Schiff staining for mucus and Gordon and Sweet Silver staining for reticulin fibres were performed as reported previously ^1^, using Harris’ haematoxylin and neutral red counterstains, respectively. Sections were visualised and photographs taken using a DM2500 microscope and LAS v4.7.1 software (Leica Microsystems). For mucus analysis, all airways on a section (minimum five) were examined and scored as having high levels of mucus if ≥ 50 % of epithelial cells in the airway were PAS^+^. The percentage of mucus^hi^ airways was reported per mouse. For reticulin analysis, four airways were analysed over the full area of the section, with priority given to round, complete airways that were not partially obscured by the sectioning process. Thickness of continuous reticulin around bronchioles was measured at 8-10 equally spaced positions around the airway using the straight line measurement tool in ImageJ. Areas immediately adjacent to a blood vessel or another airway were not measured to as not to include reticulin around these structures in measurements. Mean thicknesses were calculated per airway and the mean of all four airways was reported per mouse. All histological analysis was performed while blinded to experimental group and genotype.

**Enzyme linked immunosorbant assays (ELISAs)**

IL-13, CCL1, CCL2, CCL6, CCL8, CCL11, CCL12, CCL17, CCL24 and MIP-1γ were quantified using commercially available sandwich ELISA kits (Ready-Set-Go kit from eBioscience, Thermo Fisher for IL-13; Duoset kits from R&D Systems for the others). Total IgE and IgG1 were quantified in serum using custom sandwich ELISAs by coating high-binding plates in purified capture antibodies (IgE: clone R35-72; IgG1: clone A85-3, both Becton Dickinson) in 0.1 M sodium bicarbonate buffer, blocking plates with 3 % bovine serum albumin (BSA, Sigma-Aldrich), incubating with serum or purified antibody standards (IgE: clone C38-2; IgG1: clone MOPC-31C, both Becton Dickinson) diluted in 1 % BSA + 0.05 % Tween 20 and detecting with biotinylated detection antibodies (IgE: clone R35-118; IgG1: A85-3, both Becton Dickinson) and streptavidin-HRP (R&D Systems), also diluted in 1 % BSA + 0.05 % Tween. HDM-specific IgE and IgG1 were quantified using a modification of the total immunoglobulin method, coating plates with 50 ug/ml HDM instead of capture antibody and plating 12-point serial dilutions for each serum sample. All ELISAs were developed with TMB substrate (eBioscience, Thermo Fisher), stopped using 0.18 M sulphuric acid and absorbance values at 450 nm determined using a SpectraMax i3X plate reader (Molecular Devices). Unknown values were calculated by interpolation of absorbance values into standard curves using SoftMax Pro 7 software (Molecular Devices), except for specific immunoglobulin assays, for which area under curve values were calculated for absorbance versus dilution factor for each sample using Prism v8 (GraphPad).

**Multiplex chemokine screening**

Equal volumes of BAL fluid from each of three mice were pooled per experimental group and tested with the Proteome Profiler 25-plex Mouse Chemokine Array (R&D Systems) as per manufacturer’s instructions. Chemiluminescent images of array blots were captured using a MyECL imager (Thermo Fisher) and spot intensity analysed using Fiji/ImageJ by background correction of images and integrated density measurements of circular selections. Means of duplicate spots were calculated for each protein.

**Soluble collagen measurement**

Newly synthesised soluble collagen was quantified in lung homogenates by incubating 50 μl of homogenate or bovine collagen I standard with 1 ml of Sircol Dye Reagent for one hour at room temperature, before determining collagen concentration according the Sircol soluble collagen assay protocol (all Biocolor). Concentrations were determined by reading absorbance at 555 nm using the SpectraMax i3X plate reader and interpolation of absorbance values into bovine collagen I standard curves using SoftMax Pro 7 software.

**Flow cytometry and fluorescence activated cell sorting**

When staining for intracellular cytokines in lymphocytes, cell suspensions were incubated for four hours at 37 ^o^C in cRPMI containing 20 ng/ml phorbol 12-myristate 13-acetate (Sigma-Aldrich), 1.5 μg/ml ionomycin free acid (Merck) and 5 μg/ml Brefeldin A (Sigma-Aldrich), prior to all staining. Cell suspensions (up to 2 x10^6^ cells) were stained with Fixable Blue or Near-IR Live/Dead Stain (Invitrogen, Thermo Fisher) according to manufacturer’s instructions, before staining with antibodies to extracellular antigens in staining buffer (1 % BSA in PBS containing 0.05 % sodium azide) in the presence of CD16/CD32 blocking antibody 2.4G2 Fc Shield (TONBO Biosciences). Staining was performed at 4 ^o^C for 20 minutes, except for chemokine receptor staining, which was performed at room temperature for one hour. Extracellular anti-mouse antibodies (clone) were: PE Siglec F (E50-2440) and Brilliant Violet 711 MerTK (108928) from Becton Dickinson; Alexa Fluor 700 CD38 (90) from eBioscience, Thermo Fisher; FITC CD103 (2E7), FITC GL7 (GL7), PERCP/Cy5.5 I-A/I-E (M5/114.15.2), PERCP/Cy5.5 CD45 (30-F11), PERCP/Cy5.5 CD44 (IM7), PERCP/Cy5.5 CD11b (M1/70), Brilliant Violet 421 CD64 (X54-5/7.1), Brilliant Violet 421 CXCR5 (L138D7), Brilliant Violet 510 Ly6G (1A8), Brilliant Violet 510 CD45, (30-F11), Brilliant Violet 510 Ly6C (HK1.4), Brilliant Violet 605 CD90.2, (53-2.1), Brilliant Violet 605 CD19 (6D5), Brilliant Violet 605 NKp46 (29A1.4), Brilliant Violet 605 CD8a (53-6.7), Brilliant Violet 711 CD45 (30-F11), Brilliant Violet 711 CCR8 (SA214G2), Brilliant Violet 785 CCR2 (SA203G11), APC CD11b (M1/70), APC CD4 (RM4-5), APC CD19 (6D5), APC TCR β (H57-597), APC TCR γ/δ (GL3), APC TER-119 (TER-119), APC GR1 (RB6-8C5), APC F4/80 (BM8), APC CD11c (N418), APC FCεR1 (MAR-1), APC CD5 (53-7.3), Alexa Fluor 647 CD169 (3D6.112), Alexa Fluor 700 Ly6C (HK1.4), Alexa Fluor 700 CD45 (30-F11), Alexa Fluor 700 CD11b (M1/70), APC/Cy7 CD11c (N418), APC/Cy7 CD3e (145-2C11), APC/Cy7 CD4 (RM4-5), PE/Dazzle 594 CD86 (GL-1), PE/Dazzle 594 PD-L2 (TY25), PE/Dazzle 594 NKp46 (29A1.4), PE/Cy7 F4/80 (BM8), PE/Cy7 CD3e (145-2C11) and PE/Cy7 PD-1 (RPM1-30) from BioLegend.

Stained cells were washed twice in staining buffer and fixed in either eBioscience IC fixation buffer, or eBioscience Fixation and Permabilization buffer when subsequently staining for transcription factors (both Thermo Fisher). When required, cells were permeabilised after fixation by incubating for five minutes in Permeabilization buffer (eBioscience Thermo Fisher), stained with antibodies to intracellular antigens in Permeablization buffer at 4 ^o^C for 20 minutes and washed twice prior to analysis. Intracellular anti-mouse antibodies (clone) were: PERCP-eFluor 710 GATA3 (TWAJ), PE IL-13 (eBio13A) and PE FoxP3 (FJK-16s) from eBioscience, Thermo Fisher and Brilliant Violet 421 IL-5 (TRFK5) and APC IL-10 (JES5-16E3) from BioLegend. Data were acquired on an LSRFortessa with FACSDiva Software (both Becton Dickinson).

Flow cytometry data were analysed using FlowJo v10 software (Tree Star, Inc). In some experiments, geometric mean fluorescence intensities (gMFIs) or side scatter values were calculated and normalised by calculating fold changes from the median value obtained in the relevant control group or expressed as ΔgMFI by subtracting the gMFI obtained with the relevant fluorescence minus one control, as detailed in the respective figure legends. In some analyses, an expression index was calculated for fluorescence parameters by multiplying gMFI values of the positive population by the proportion of positive cells (0-1), prior to calculation of fold changes from the median of the control group.

Fluorescence-activated cell sorting of mouse BAL cells was performed on a FACSAria instrument with a 100 μm nozzle (Becton Dickinson), with live cells determined by exclusion of ToPro3 iodide viability dye (Invitrogen, Thermo Fisher). SigF^+^ AM-like cells and SigF^-^ MPs were sorted as ToPro3^-^ CD45^+^ lineage (CD19, CD90.2, NKp46)^-^ CD64^+^ CD11c^+^ cells positive or negative, respectively, for Siglec F. Ly6C^hi^ monocytes were sorted as ToPro3^-^ CD45^+^ lineage^-^ CD11c^-^ Siglec F^-^ CD11b^+^ Ly6C^hi^. Purity checks showed mean post-sort purities of 86, 93 and 97 %, respectively, for Sig F^+^ AM-like cells, SigF^-^ MPs and Ly6C^hi^ monocytes. Sorted cells were applied to glass microscope slides using a Shandon Cytospin 3 and stained using Shandon Kwik Diff (both Thermo Fisher) as per manufacturer’s instructions, for visualisation of morphology.

**RNA extraction and cDNA conversion**

RNA from whole mouse lung tissue was obtained by homogenising right sub-caval lung lobes in 500 μl RLT buffer (QIAGEN) supplemented with 1 % β-mercaptoethanol (Sigma-Aldrich), using the FastPrep 24 bead homogeniser and Lysing Matrix D, centrifuging homogenates at 11 000 x *g* for ten minutes at 4 ^o^C to remove debris and extracting RNA from 350 ul of the resultant supernatants using the RNeasy Plus Mini Kit (QIAGEN), according to manufacturer’s instructions. 1 μg of whole lung RNA was converted to cDNA using the High Capacity cDNA conversion kit, without RNase inhibitor (Applied Biosystems, Thermo Fisher). RNA from flow cytometry-sorted mouse cells and adhesion-enriched AM layers from mouse BAL was obtained by lysing cells in 350 ul RLT + 1% β-mercaptoethanol and extracting RNA from lysates using the RNeasy Plus Micro Kit (QIAGEN), according to manufacturer’s instructions. Up to 100 ng of RNA from these cells was converted to cDNA using the GoScript Reverse Transcriptase System with random primers (Promega). Bead-sorted human AMs were lysed in TRIZol reagent (Invitrogen, Thermo Fisher), which was mixed 5:1 with chloroform and centrifuged at 10 000 *g* for ten minutes at 4 ^o^C to obtain RNA in aqueous layers. RNA was then extracted from the aqueous layer by mixing with an equal volume of 70 % ethanol and using the RNeasy Mini Kit with on-column DNase digest (both QIAGEN), as per manufacturer’s instructions. RNA integrity was confirmed to be above 8.0 following extraction using the TapeStation 2200 and RNA Screen Tapes (both Agilent) ahead of sequencing library preparation.

**Quantitative PCR**

Duplicate 6 μl volume TaqMan Fast Advanced reactions were performed using TaqMan Fast Advanced Master Mix and off-the-shelf FAM-labelled primer/probe assays (all Applied Biosystems, Thermo Fisher). The following mouse-specific assays were used (unique identifier): *Actb* (Mm00607939_s1), *Gapdh* (Mm99999915_g1), *Tgfb1* (Mm00441724_m1)*, Pparg* (Mm00440940_m1)*, Chil3* (Mm00657889_mH)*, Nos2* (Mm00440502_m1), *Il4* (Mm00445259_m1), *Il5* (Mm00439646_m1), *Ccl2* (Mm00441242_m1)*, Ccl6* (Mm01302419_m1)*, Ccl7* (Mm00443113_m1)*, Ccl8* (Mm01297183_m1), mouse *Ccl9* (Mm00441260_m1), *Ccl12* (Mm01617100_m1), *Il1a* (Mm00439620_m1), *Il1b* (Mm00434228_m1), *Il6* (Mm00446190_m1), *Il10* (Mm01288386_m1), *Il12a* (Mm00434169_m1), *Il12b* (Mm01288989_m1), *Il23a* (Mm00518984_m1) and *Tnf* (Mm00443258_m1). Reactions were run on a Viia™ 7 instrument, using Viia™ 7 Software to calculate threshold cycle (Ct) values (both Applied Biosystems, Thermo Fisher). Means of Ct values from *Gapdh* and *Actb* housekeeping control genes were used as normalisation factors for each sample. Expression of each gene was determined as 2^-(Ct-normalisation factor)^ and fold changes from median values of the appropriate negative control group calculated where necessary.

**Sequencing library preparation and RNA sequencing**

200 ng RNA per sample was polyA-purified and converted to cDNA sequencing libraries using the Illumina TruSeq Stranded mRNA v2 kit as per manufacturer’s instructions, including addition of unique indexes to each sample to allow multiplexing. Libraries were sequenced (110 base pair, paired end reads) on an Illumina HiSeq 4000 system and data de-multiplexed and adapter sequences removed prior to analysis. FASTQC analysis confirmed Phred scores to be >30 across reads for all samples.

**RNA-Seq analysis**

Reads were aligned to the human transcriptome (Ensembl Hg38, 2016.12.01) using the COBWeb aligner within the StrandNGS analysis tool (Strand LS). A minimum 90 % sequence identity and match length of 25 base pairs, with a maximum 5 % sequence identity gap and maximum one novel splice event, were required for reads to align to a transcript. > 70 % of reads were successfully aligned in all samples. Transcript expression values were quantified and Log_2_ normalised from aligned reads using the DESeq method within StrandNGS. Low-abundance transcripts for which five or more raw reads were not detected in at least 25 % of samples were removed prior to further analysis.

**Supplementary references**

1. Saglani S, Mathie SA, Gregory LG, Bell MJ, Bush A, Lloyd CM. Pathophysiological features of asthma develop in parallel in house dust mite-exposed neonatal mice. American Journal of Respiratory Cell and Molecular Biology 2009; 41:281-9.

**Table E1**

| **Subject**  **(Diagnosis)** | **Age/y** | **Sex** | **Mac**  **%** | **Neu**  **%** | **Eos**  **%** | **Lym**  **%** | **IgE**  **(IU/ml)** | **sIgE**  **(Y/N)** | **Viral**  **PCR** | **Bacterial culture** | **ABX** | **ICS** | **Iβ_2_A** | **LTRA** |
| --- | --- | --- | --- | --- | --- | --- | --- | --- | --- | --- | --- | --- | --- | --- |
| 18  (chronic cough) | 12.17 | M | 79.1 | 2.74 | 1.77 | 12.5 | 271 | Y | - | - | N | N | N | N |
| 20  (chronic cough) | 16.0 | F | 85.1 | 8.23 | 0.61 | 1.2 | 454 | Y | *RV* | - | Y | N | N | N |
| 21  (chronic cough) | 4.92 | F | 73.9 | 5.87 | 1.98 | 12.3 | 66 | N | - | - | N | N | N | Y |
| 22  (chronic cough) | 9.50 | F | 64.3 | 6.5 | 1 | 20 | 2673 | Y | - | - | N | N | N | N |
| 23  (chronic cough) | 3.67 | F | 76.2 | 4.3 | 0.64 | 15.9 | 28 | N | - | - | N | N | N | N |
| **Chronic cough**  Median (IQR)  or % | 9.50 (9.79) | 80% F | 76.2  (13) | 5.87 (3.85) | 1  (1.25) | 12.5 (11.2) | 271 (1517) | 60% Y | 20% +ve | 0% +ve | 20% Y | 0% Y | 0% Y | 20% Y |
| 14  (severe wheeze) | 4.92 | M | 25.3 | 50.3 | 0.75 | 11.47 | 21 | N | - | *Mc*, *Sp* | N | Y | Y | Y |
| 15  (severe wheeze) | 4.58 | F | 33.4 | 19.8 | 0 | 39.2 | 10 | N | *RV* | *Mc* | N | Y | Y | Y |
| 16  (severe wheeze) | 2.50 | F | 19 | 7.2 | 2.5 | 60 | 1919 | Y | *BV* | - | N | Y | Y | Y |
| **Severe wheeze**  Median (IQR)  or % | 4.58 (2.42) | 67% F | 25.3 (14.4) | 19  (43.1) | 0.75 (2.5) | 39.2  (48.5) | 21 (1909) | 33.3% Y | 66.7% +ve | 66.7% +ve | 0% Y | 100% Y | 100% Y | 100% Y |

**Supplementary table legends**

**Table E1. Demographic, clinical immunology and medication data for subjects in the paediatric AM RNA-Seq study**

IQR, interquartile range; F, female sex; M, male sex; Mac, BAL macrophages; Neu, BAL neutrophils; Eos, BAL eosinophils; Lym, BAL lymphocytes; sIgE, allergen-specific IgE to ≥1 aeroallergen; Y/N, yes/no; *RV*, rhinovirus; *BV*, bocavirus; *Mc*, *Moraxella catarrhalis*; *Sp*, *Streptococcus pneumoniae*; (-) indicates a negative result. ABX, prophylactic antibiotics; ICS, inhaled corticosteroids; Iβ_2_A, inhaled β_2_ agonist; LTRA, leukotriene receptor antagonist*.*

**Supplementary figure legends**

**Figure E1. Mononuclear phagocyte identification and analysis in neonatal *Tgfb1*^ΔCD11c^ mice and paediatric human BAL**

**A-H**) Lung cells from *Tgfb1*^ΔCD11c^ and *Tgfb1*^fl/fl^ mice were analysed by flow cytometry at the indicated ages. **A**) Myeloid cell gating strategy. Lineage cocktail: CD19, CD90.2 and NKp46; BV, Brilliant Violet; AF, Alexa Fluor; IMs, interstitial macrophages; monos, monocytes; cDC, conventional dendritic cell. This strategy was used for the indicated populations throughout the study, unless otherwise specified. **B-D**) AM counts (**B**), geometric mean fluorescence intensity of Siglec F staining relative to *Tgfb1*^fl/fl^ controls (**C**) and percentage of CD11b^+^ AMs (**D**) in adult (8–10-week-old) mice. **E-G**) Parameters as in (**B-D**), in 8-10-month-old mice. **H**) *Tgfb1* qPCR relative gene expression in AMs isolated from P14 *Tgfb1*^ΔCD11c^ and *Tgfb1*^fl/fl^. **I**) Numbers of the indicated MP populations in lungs of P28 and adult mice. **J**) Representative plot showing post-sort paediatric human AMs. Plot is from a child with chronic cough. Data in (**B-H**) show means and individual replicate values and are pooled from analysis of a minimum of two litters. Statistical results shown are from Student’s unpaired t-tests; *, *P* < 0.05; ****, *P* < 0.0001; ns, non-significant.

**Figure E2. Mononuclear phagocyte phenotypes in *Tgfb1*^ΔCD11c^ mice following neonatal HDM exposure**

**A**) Numbers of lung SigF^+^ AM-like cells (Siglec F^+^ CD11c^+^ CD64^+^ F4/80^+^), SigF^-^ MPs (Siglec F^-^ CD11c^+^ CD64^+^ F4/80^+^), total CD11c^+^ CD64^+^ F4/80^+^ MPs, Ly6C^hi^ monocytes (CD11c^-^ Siglec F^-^ CD11b^+^ Ly6C^hi^) and interstitial macrophages (CD64^+^ F4/80^+^ CD11c^-^ Siglec F^-^ CD11b^+^ Ly6C^-^), determined by flow cytometry. **B**) Sorting strategy for MP populations from BAL of HDM-treated mice. Representative plots from a *Tgfb1*^ΔCD11c^ mouse. Lineage cocktail: CD19, CD90.2, NKp46 and Ly6G. **C**) Representative histograms showing CD11b expression on BAL MPs. **D**) Percentages of BAL MP populations positive for CD11b or MHC-II. **E**) Representative histograms showing surface marker expression on BAL MP populations. **F**) Geometric mean fluorescence intensity of CD169 and PD-L2 on BAL SigF^+^ AM-like cells positive for these markers. **G**) Relative qPCR gene expression in sorted BAL MPs. Scatter/bar plots show means or medians of data pooled from 2-4 individual experiments with all individual replicates. Statistical results are from Kruskal-Wallis tests with Dunn’s *post hoc* test for multiple comparisons and either Student’s unpaired t-tests or Mann-Whitney U tests for single comparisons. *, *P* < 0.05; **, *P* < 0.01; ***, *P* < 0.001; ****, *P* < 0.0001; ns, non-significant.

**Figure E3. Additional data on mononuclear phagocytes and monocyte chemokine expression in *Tgfb1*^ΔCD11c^ mice**

**A**) Numbers of the indicated MP populations in lungs from CCR2 blocking experiments, determined by flow cytometry. **B**) Concentrations of CCL6 and MIP-1𝛾 in lung homogenates of *Tgfb1*^ΔCD11c^ and *Tgfb1*^fl/fl^ mice, determined by ELISA. **C-D**) Relative qPCR gene expression in MP populations sorted from BAL of HDM-treated mice (**C**) or AMs isolated from BAL of naïve P14 mice (**D**). Scatter/bar plots show data pooled from two individual experiments and show means or medians with all individual replicates. Statistical results shown are from Kruskal-Wallis tests with Dunn’s *post hoc* test for multiple comparisons and either Student’s unpaired t-tests or Mann-Whitney U tests for single comparisons. *, *P* < 0.05; **, *P* < 0.01; ***, *P* < 0.001; ****, *P* < 0.0001; ns, non-significant.

**Figure E4. Allergic airway disease in HDM-treated *Tgfb1*^ΔCD11c^ mice**

**A**) CD4 T cell and ILC gating strategy, starting with live, singlet, CD45^+^ cells. ILC lineage cocktail: CD11b, TCRβ, TCRγ/δ, CD19, CD5, F4/80, GR-1, CD11c, TER-119 and FcεRI. **B**) Numbers of neutrophils determined by flow cytometry. **C**) Fold changes in relative qPCR gene expression in lungs. **D**) Lung CD4 T cell subset numbers. **E**) Cytokine-positive CD4 T cell frequencies in mLNs. **F**) Lung resistance at increasing methacholine concentrations ([MCh]). **G**) HDM-specific immunoglobulin ELISA absorbance values from individual replicate mice (N=7 *Tgfb1*^fl/fl^ and N=11 *Tgfb1*^ΔCD11c^). **H**) Gating strategy for T follicular helper cells (Tfh) and germinal centre (GC) B cells, starting with live, singlet, CD45^+^ lymphocytes. **I**) Frequencies of mLN Tfh and GC B cells. **J**) PAS airway mucus scoring and representative images. Scale bar = 200 μm. Data are pooled from 2-4 individual experiments, except for IL-10^+^ in (**D**) and data in (**E**), which represent one of two experiments. Scatter/bar plots show means or medians with individual replicates. (**F**) shows means ±SEM of N=11 *Tgfb1*^fl/fl^/PBS, N=5 *Tgfb1*^ΔCD11c^/PBS, N=14 *Tgfb1*^fl/fl^/HDM and N=15 *Tgfb1*^ΔCD11c^/HDM). Statistical results are from Student’s unpaired t-tests or Mann-Whitney U tests. *, *P* < 0.05; **, *P* < 0.01; ****, *P* < 0.0001; ns, non-significant.

**Figure E5. Comparable conventional DC frequency and maturation marker expression in HDM-treated *Tgfb1*^ΔCD11c^ and *Tgfb1*^fl/fl^ control mice**

Flow cytometry analysis of cDC populations. **A** and **C**) Numbers of cDC1s (**A**) and cDC2s (**C**) in lungs of PBS- or HDM-treated mice. **E** and **G**) Frequencies of cDC1s (**E**) and cDC2s (**G**) in mLNs of HDM-treated mice. (**B**) and (**D**) show CD86 and MHC-II expression in lung tissue and (**F**) and (**H**) show equivalent data from mLNs. CD86 and MHC-II expression are presented as relative staining index and geometric mean fluorescence intensity, respectively, with representative histograms for each. Scatter/bar plots show means or medians of data pooled from 2-4 experiments, with individual replicates. Statistical results shown are from a Mann Whitney U test (**C**) and Student’s unpaired t-test (**D**); ns, non-significant.

**Figure E6. Anti-CCR2 therapeutic effects in HDM-treated *Tgfb1*^ΔCD11c^ mice**

**A-B**) Flow cytometry quantification of numbers of cytokine-positive lung CD4 T cells (**A**) and BAL and lung eosinophils (**B**). **C**) Representative histograms showing a lack of CCR2 surface staining on eosinophils. Ly6C^hi^ monocytes are shown as a positive control. Scatter/bar plots show means or medians of data pooled from two experiments, with individual replicates. Statistical results shown are from Kruskal-Wallis with Dunn’s *post hoc* test for multiple comparisons, or one-way ANOVA with Sidak’s *post hoc* test for multiple comparisons. *, *P* < 0.05; **, *P* < 0.01; ****, *P*<0.0001. ns, non-significant.

**Figure E7. Proinflammatory cytokine and chemokine expression in HDM-treated *Tgfb1*^ΔCD11c^ mice**

**A**) Fold changes in relative qPCR gene expression in lungs. **B**) Chemiluminescent images of chemokine array immunoblots of BAL from HDM-treated mice, with chemokines of interest highlighted. **C-F**) Chemokine concentrations determined by ELISA. (**B**) shows results from a single assay pooling BAL from three mice per group. Scatter/bar plots show means or medians of data pooled from two experiments, with individual replicates. Statistical results shown are from either Student’s unpaired t-tests or Mann-Whitney U tests for single comparisons. *, *P* < 0.05; **, *P* < 0.01; ns, non-significant.

**Figure S8. Additional data on CCR8 expression and blockade in HDM-treated *Tgfb1*^ΔCD11c^ mice**

**A**) Δgeometric mean fluorescence intensity (gMFI - gMFI of fluorescence minus one control) of CCR8 staining on total and IL-13^+^ (after PMA/ionomycin stimulation) CD4 T cells and ILCs, from HDM-treated mice. Analysis of CCR8 blocking (αCCR8) experiments. **B**) Numbers of lung GATA3^+^ ILCs. **C**) Concentration of IL-13 in lung tissue determined by ELISA. **D**) Numbers of BAL eosinophils. **E**) Concentrations of total IgE and IgG1 in serum ~~as~~ determined by ELISA. **F-H**) Numbers of the indicated MP populations in BAL or lung (**F-G**) or proportions in peripheral blood (**H**). Scatter/bar plots show means or medians of data pooled from two experiments, with all individual replicates. Statistical results shown are from Kruskal-Wallis with Dunn’s *post hoc* test for multiple comparisons and either Student’s unpaired t-tests or Mann-Whitney U tests for single comparisons. *, *P* < 0.05; ***, *P* < 0.001; ****, *P*<0.0001. ns, non-significant.
